# Supplementary material for: The dynamics and geometry of choice in the premotor cortex
Source: Nature. 2025 Jun 25;645(8079):168–76. doi: 10.1038/s41586-025-09199-1 (PMC12408350; doi:10.1038/s41586-025-09199-1)
Supplement: Supplementary file 1 — Supplementary Notes, Supplementary Methods and Supplementary Figs 1–9. [file 41586_2025_9199_MOESM1_ESM.pdf]

nature portfolio

https://doi.org/10.1038/s41586-025-09199-1

Supplementary information

The dynamics and geometry of choice in the premotor cortex

In the format provided by the authors and unedited

Supplementary Information for: The Dynamics and Geometry of Choice in the Premotor Cortex

Mikhail Genkin,[1] Krishna V. Shenoy,[2,3]
Chandramouli Chandrasekaran,[4,5,6] Tatiana A. Engel[1,7*]

[1]Cold Spring Harbor Laboratory, Cold Spring Harbor, NY, USA,
[2]Howard Hughes Medical Institute, Stanford University, Stanford, CA
[3]Department of Electrical Engineering, Stanford University, Stanford, CA
[4]Department of Anatomy & Neurobiology, Boston University, Boston, USA
[5]Department of Psychological and Brain Sciences, Boston University, Boston, USA
[6]Center for Systems Neuroscience, Boston University, Boston, MA
[7]Princeton Neuroscience Institute, Princeton University, Princeton, NJ
*Corresponding author e-mail: tatiana.engel@princeton.edu.

May 17, 2025

Contents

1 Supplementary Notes **2**
1.1 The duality between neural tuning and population geometry . . . 2
1.2 Validation of the inference framework in experimental data . . . 3
1.3 Dimensionality of neural manifolds . . . 5
1.4 Relationship to other models of latent neural dynamics . . . 6
1.5 Population dynamics and geometry on correct and error trials . . . 8

2 Supplementary Methods **9**
2.1 Analytical calculation and numerical evaluation of the likelihood . . . 9
2.2 Analytical derivation of the likelihood derivatives . . . 12
2.3 Maximum likelihood optimization with ADAM algorithm . . . 15
2.4 Model selection . . . 16
2.5 Viterbi algorithm . . . 17

3 Supplementary References **18**

4 Supplementary Figures **21**

1 Supplementary Notes

1.1 The duality between neural tuning and population geometry

Tuning curves and population geometry provide dual views on the same neural responses: tuning curves at the level of single neurons, and population geometry at the level of population activity patterns [1]. To understand this duality, consider time-averaged responses of N neurons to K static stimuli, arranged in an $N \times K$ neural response matrix (Extended Data Fig. 1a). The columns of this matrix define the neural population state space, where each axis represents activity of one neuron and each point represents a stimulus, collectively forming the population geometry. The rows of the same matrix define the tuning curves of individual neurons to the stimuli. The neural response matrix is fully determined by specifying either all of its columns or all of its rows. Hence, the tuning curves of all neurons in the population uniquely define the geometry of stimulus representation in the neural population state space.

While the duality between neural tuning and population geometry is well established for encoding of sensory stimuli [1], analogous frameworks have been lacking for the representation of dynamic cognitive variables that transiently unfold over time in single-trial neural activity. Conventionally, neural responses during cognitive tasks are characterized with respect to static external task variables, e.g., stimulus coherence or choice (Extended Data Fig. 1b). However, single neurons have complex temporal response profiles, such that their selectivity for static external variables often changes significantly over time [2, 3, 4]. For example, a neuron may show higher firing rate for left choices early in the trial but switch its preference to right choices toward the trial end (Extended Data Fig. 1b). The temporal inconsistency of neural tuning/selectivity for static external task variables led to a widespread view that cognitive computations arise from complex population dynamics, while tuning/selectivity of single neurons is not interpretable [3, 4].

Contrasting this view, we propose and test a novel hypothesis which unifies the neural encoding of sensory stimuli and dynamic cognitive variables. Rather than selectivity for static external task variables, we consider neural tuning to internally generated dynamic variables that implement cognitive computations (Extended Data Fig. 1c). These tuning functions uniquely specify the representational geometry of the internal dynamic variable in the population state space, precisely as they do for sensory stimuli. For example, the value of the decision variable at any given time is encoded by the position of population activity along a one-dimensional manifold in the population state space (Extended Data Fig. 1c). The population trajectories traverse this manifold as they evolve toward one or another choice on each trial. The geometry of these population trajectories is uniquely specified by tuning functions of single neurons to the decision variable, with the exact same dual relationship between neural tuning and population geometry as for sensory stimuli. In addition, the dynamics model captures the single-trial dynamics of the decision variable, which govern time evolution of population activity along the manifold on single trials.

While neural response properties arise from circuit dynamics governed by recurrent connectivity [5], neither tuning curves nor population geometry is inherently closer to this mechanistic origin. Population geometry does not generate tuning curves, nor do tuning curves generate population geometry. Neither has a causal relationship to the other; both are merely equivalent descriptions of the same data.

1.2 Validation of the inference framework in experimental data

While we demonstrated the accuracy of our nonparametric inference framework on synthetic data with known ground truth, biological data present a unique challenge since the ground truth is typically unknown. Causal optogenetic perturbations offer a powerful method for testing competing hypotheses about neural dynamics, serving as a ground truth for evaluating latent dynamical models. Therefore, we further validated our modeling approach using a publicly available dataset, where the ground truth has been established through causal optogenetic perturbations [6, 7].

In this experiment, population spiking activity was recorded from the anterior lateral motor cortex (ALM) in mice performing a delayed response auditory discrimination task [7]. The study used bilateral optogenetic perturbations in ALM to distinguish between three alternative hypotheses about the mechanism of working memory maintenance during the delay period: line attractor, single attractor, or multistable discrete attractor dynamics. The observed recovery patterns of trial-averaged neural responses following optogenetic perturbations were consistent with the multistable discrete attractor hypothesis. Although this study did not analyze neural dynamics on single trials, the perturbation results establish multistable discrete attractor dynamics as the ground truth during the memory delay period of the task. We therefore tested whether our modeling approach could correctly identify multistable attractor dynamics from the observational data alone, without using perturbation data for model fitting.

We analyzed population spiking activity recorded from ALM during the random delay task [7]. In brief, mice discriminated the frequency of an auditory tone and reported their decision with a left or right lick after a delay period, with durations randomized across trials. At the start of each trial, an auditory tone of one of two possible frequencies was presented for a 1.15 s sample period, instructing a left or right choice. Accordingly, there were two stimulus conditions: left stimulus and right stimulus. After the tone, mice had to wait for a variable-duration delay period until an auditory go cue prompted them to report their choice. Delays in each trial were randomly sampled from eight durations (0.3, 0.5, 0.7, 0.9, 1.2, 2, 3.2 and 4 s) approximating the exponential distribution with 0.2 s offset. Randomized delays prevented the mice from predicting the timing of the lick response. On 25% of the trials with 2 s delay duration, bilateral ALM photoinhibition was deployed from the start of the delay period for 0.6 s, followed by a 0.4 s ramping down period of photoinhibition [7].

We fitted our model to population spiking activity during the delay period to identify single-trial dynamics supporting working-memory maintenance. We fitted the model exclusively to trials without photoinhibition, combining all correct and error trials, while excluding trials on which the mouse did not respond or responded before the go cue. We used population spiking activity from the start of the delay period until the go cue onset on each trial for model fitting. In each session, we selected neurons for the analysis based on two criteria: a sufficiently high trial-averaged firing rate during the delay (exceeding ~ 15 Hz in at least one stimulus condition) and a substantial difference in trial-averaged firing rates between the left and right stimulus conditions. We included sessions that had at least 6 simultaneously recorded neurons satisfying both criteria, yielding 6 sessions out of 24 sessions that included photoinhibition trials. Each of the analyzed sessions contained 6 to 7 single neurons that met our selection criteria.

We fitted the model to all available trials, restricting the potential $\Phi(x)$ and tuning functions $f_i(x)$ to be the same in the left and right stimulus conditions, while allowing the initial state distribution $p_0(x)$ to differ between stimulus conditions. We enforced a single shared potential $\Phi(x)$

because no external stimulus is present during the delay period to induce distinct dynamics in each stimulus condition. On the other hand, the initial state distribution $p_0(x)$ can be different between the left and right stimulus trials, because stimulus-selective inputs during the sample period can drive the ALM activity to different initial states at the delay onset. The noise magnitude was fixed at $D = 0.5$.

Since the delay duration on each trial was controlled by the experimenter independently of the neural dynamics, we fitted the model with reflecting boundary conditions [8]. Under reflecting boundary conditions, the trajectories are reflected back into the latent domain whenever they reach a boundary, and no constraints are imposed on the latent state at the end of the delay. We selected the optimal model based on feature consistency (Supplementary Methods 2.4).

In an example session, the inferred potential shape exhibited three wells separated by barriers (Extended Data Fig. 4a), consistent with the multistable discrete attractor hypothesis as was demonstrated using optogenetic perturbations [7]. The inferred initial state distribution $p_0(x)$ was peaked near the left attractor on the left stimulus trials, and near the right attractor on the right stimulus trials, while also carrying substantial weight near the middle attractor (Extended Data Fig. 4a). This distribution of initial states suggests that the left and right attractors correspond to the working memory of the left and right choices, respectively, and the middle attractor is an intermediate state. Tuning functions were diverse across single neurons (Extended Data Fig. 4b).

To further test the correspondence between the attractor states and the animal's upcoming choices, we used the fitted model to decode latent trajectories $x(t)$ from spike data on single trials (Extended Data Fig. 4c, Supplementary Methods 2.5). On most correct trials, the population trajectories stayed near the attractor corresponding to the correct choice for nearly the entire delay period. A considerable fraction of these trajectories started at the middle attractor, transitioning to the correct-choice attractor early in the delay, indicating that the middle attractor may represent the nonselective baseline state at the trial start. On only a handful of correct trials, the trajectories resided near the incorrect choice attractor for the entire delay duration. Accordingly, the distribution of the latent state at the end of the delay period was sharply peaked at the attractor corresponding to the correct choice. The opposite pattern was observed on error trials: most trajectories either remained near the middle attractor through the entire delay, or transitioned among all three attractors. As a result, the distribution of the latent state at the end of the delay peaked near the middle and incorrect choice attractors on error trials.

Our model enables interpreting optogenetic perturbation effects on neural dynamics across time on single trials, extending beyond the analysis of recovery patterns in trial-averaged responses [7]. We used the model fitted to data from unperturbed trials to decode single-trial latent trajectories $x(t)$ from spike data on photoinhibition trials (Extended Data Fig. 4d). During photoinhibition (0–600 ms from delay onset), latent trajectories tended to converge toward the middle attractor, further supporting its interpretation as a nonselective baseline state. As photoinhibition was ramping down (600–$1,000$ ms from delay onset), the trajectories transitioned to the correct-choice attractor on correct trials, whereas on error trials, they either shifted to the incorrect-choice attractor or remained trapped at the middle attractor. The distributions of latent states at the end of the delay were similar between trials with and without photoinhibition, both for correct and error trials. Overall, single-trial dynamics were similar between trials with and without photoinhibition, with perturbations causing a transient, time-aligned shift in the network state.

Our model discovered multiple discrete attractors in all fitted sessions (three attractor wells: 4 out of 6 sessions, 67%; four attractor wells: 2 out of 6 sessions, 33%). The inferred initial state

distributions and decoded single-trial dynamics were also consistent across all sessions: the two outer attractors corresponded to the left and right choices on correct trials, while intermediate attractors represented the baseline states.

Thus, our model correctly identified multistable discrete attractor dynamics by fitting single-trial spiking activity under normal task conditions, without using perturbation data. In contrast, trial-averaged neural activity does not uniquely determine a model of population dynamics on single trials [9]. Hence, causal perturbations are required for correctly identifying dynamics when fitting models to trial-averaged neural responses [10]. Our modeling approach yields comparable insights without using external causal perturbations by analyzing single-trial activity, which inherently contains transient deviations from the average that act as naturally occurring perturbations within the repertoire of activity patterns produced by the dynamical system [9]. These results validate the accuracy of our dynamics inference approach in biological data.

1.3 Dimensionality of neural manifolds

To establish the correspondence between the dynamics and geometry of the decision variable uncovered by our model in PMd data and the classical attractor networks [11, 12], we must consider three distinct types of dimensionality: the number of variables in a neural network model, intrinsic dimensionality of a neural manifold, and linear dimensionality of a neural manifold [13].

Number of variables in a network model. The spiking neural network model of decision-making [11] is a high-dimensional dynamical system described by $3N_E + 2N_I$ variables, which are membrane potentials and synaptic conductances of N_E excitatory and N_I inhibitory neurons [11]. Using mean-field approximation, the spiking network can be reduced to a two-dimensional dynamical system, in which the variables are the average NMDA conductances of two choice-selective excitatory populations [12]. This two-dimensional model was developed for the ease of interpreting flow fields on a phase plane, not because two is the correct number of variables to describe mean-field dynamics of the spiking network. In fact, a more accurate mean-field approximation of the spiking network includes 11 variables: AMPA and NMDA conductances of three excitatory populations (two choice-selective and one non-selective), GABA conductance of the inhibitory population, and firing rates of all four neural populations (Eqs. 3–7 in Ref. [12]).

Intrinsic dimensionality of a neural manifold. In both the spiking network model (defined by $3N_E+2N_I$ variables) and its mean-field approximation (defined by 2 variables), the trajectories arising during decision-making are confined to a nonlinear manifold with intrinsic dimensionality equal to one. Intrinsic dimensionality is the minimal number of continuous variables necessary to parameterize the manifold [5, 13]. On each trial, network trajectories start at a symmetric low-activity state and follow nearly one-dimensional stereotypic paths to reach either of the two choice attractors. When varying stimulus difficulty, the shape of these one-dimensional paths remains nearly invariant and only the speed and direction of dynamics along these paths change, consistent with our findings in the PMd data. One-dimensional trajectories traced by the network during decision-making can be parametrized by a single variable, corresponding to the decision variable in our model. Thus, tuning functions to the decision variable in our model capture the geometry of neural trajectories during decision-making and the potential describes the dynamics along these trajectories.

Although the spiking network model has a high-dimensional state space and the mean-field model has two-dimensional state space, trajectories arising during decision-making do not explore

the entire state space of these dynamical systems. In principle, appropriately designed inputs can drive the system to any corner in its state space. For example, a strong excitatory input delivered to both choice-selective populations can stabilize a symmetric high-activity state. However, such inputs do not occur during normal decision-making behavior and the corresponding regions of the two-dimensional state space are never visited. As a result, the intrinsic dimensionality of the neural activity manifold is lower than the dimensionality of the network state space.

Linear dimensionality of a neural manifold. The tuning functions in our model describe how firing rates of single neurons change along the trajectories taken by the network during decision-making. Thus, the tuning functions capture the geometry of decision-making trajectories in the population state space. Since these trajectories are nonlinear, the linear dimensionality of the manifold they form is greater than one. The linear dimensionality is the smallest number of orthogonal directions that span a linear subspace containing the manifold [5, 13]. In the spiking network model, choice-selective excitatory neurons have only two types of tuning functions, hence the decision manifold they form spans two linear dimensions. In PMd, tuning functions are heterogeneous, and the manifold has higher linear dimensionality.

In summary, the spiking network model enables us to establish a mechanistic interpretation of the one-dimensional decision variable and dynamics identified in PMd by our model. In both spiking network and PMd data, the decision-related activity evolves along trajectories that form a manifold with intrinsic dimensionality close to one. This manifold is parametrized by the one-dimensional decision variable uncovered by our model. The potential governing the dynamics along this manifold shows the same signatures of the attractor mechanism in the spiking network model and PMd. However, the linear dimensionality of the manifold is higher in PMd than in the spiking network due to heterogeneous response profiles of PMd neurons.

1.4 Relationship to other models of latent neural dynamics

Our approach introduces three fundamental advances over other methods for modeling neural dynamics: (i) simultaneous inference of nonlinear tuning functions and a nonlinear latent dynamical system, (ii) flexible nonparametric inference, and (iii) principled approach for selecting the complexity of inferred model features.

Flexible nonparametric inference of latent dynamics. Most existing models of latent neural dynamics trade off flexibility for interpretability or vice versa. On one hand, flexible high-dimensional recurrent neural networks can approximate any dynamics but do not yield interpretable low-dimensional flow fields [14, 15, 16, 17, 18]. On the other hand, interpretable models, such as linear dynamical systems [19] or Hidden Markov Models (HMM) [20, 21], rely on rigid parametric assumptions about latent dynamics. These models merely approximate data with *a priori* assumed dynamics but do not allow for discovering the dynamical laws governing latent trajectories. For example, a linear dynamical system can only have a single fixed point, and an HMM approximates any trajectory as a sequence of discrete states even if the ground-truth trajectory is continuous. Such approximations may produce high data likelihood, but it does not prove that the *a priori* chosen model faithfully describes dynamics in the data. For example, if an n-state HMM accurately approximates a continuous trajectory, it does not follow that this trajectory arises from transitions among n metastable attractors.

Our framework belongs to a new class of *flexible* and *intrinsically interpretable* models, which discover the low-dimensional flow field of the latent dynamical system directly from data. Flexible

models cover a continuous space of hypotheses about latent dynamics within a single model architecture and therefore enable discovering the flow-field by fitting the model to data. The low-dimensional flow field can be approximated with a set of basis functions [22], a Gaussian process [23], or a deep neural network [24]. All these formulations have computationally intractable data likelihood and thus rely on approximate variational inference maximizing the evidence lower bound of the marginal log-likelihood. In contrast, we model the flow field explicitly as an unknown continuous function and analytically compute the functional derivative of the data likelihood with respect to this continuous function. This approach enables us to perform the exact maximum likelihood inference without variational approximations and provides an additional advantage of imposing a uniform prior over the space of continuous functions, avoiding inductive biases of the approximation schemes [22, 23, 24]. In all these models, the inferred low-dimensional flow field is immediately interpretable, in contrast to models that represent the flow field implicitly within a high-dimensional connectivity matrix of an RNN and therefore lack interpretability [14, 15].

Selecting the complexity of inferred model features. All flexible models are generally under-constrained by data and require regularization. However, choosing the optimal regularization level in flexible models to achieve correct interpretation of dynamics in the data is nontrivial [25]. The classical bias-variance tradeoff, which is the foundation of validation-based model selection, does not hold for flexible models with high capacity [26]. As a result, flexible models optimized for predictive performance on validation data often contain spurious features and cannot be reliably interpreted [25, 27]. To overcome this problem, we developed an alternative model selection strategy that identifies models with correct interpretation by comparing features of models fitted on different data samples to separate true features from noise [25]. Other methods for the flow field inference did not consider the problem of model selection and set the regularization level *ad hoc* to produce compelling results [22, 23, 24]. The *ad hoc* regularization undermines the interpretation of the inferred flow field, because the model may be underfitted or overfitted if the regularization penalizes the model complexity too much or too little, which remains unknown in the absence of diagnostic tests.

Simultaneous inference of nonlinear dynamics and tuning functions. The inference of nonlinear tuning functions simultaneously with a nonlinear latent dynamical system is unique to our study. Previous methods that inferred nonlinear tuning functions of neurons to latent variables either used a linear latent dynamical system [28] or approximated latent trajectories with a Gaussian process without modeling a dynamical system governing these trajectories [29]. These methods approximated tuning functions either with a feedforward neural network [28] or with a Gaussian process [29]. In both formulations, the data likelihood is computationally intractable, therefore these methods relied on approximate variational inference maximizing the evidence lower bound of the marginal log-likelihood. In contrast, we model tuning functions explicitly as unknown continuous functions and analytically compute functional derivatives of the data likelihood with respect to these continuous functions, which enables us to perform the exact maximum likelihood inference of all model components avoiding biases introduced by variational approximations and inductive biases of the approximation schemes.

Modeling spiking variability. Like many other methods, we model spikes of each neuron as a doubly stochastic Poisson process with an instantaneous firing rate varying as a function of the latent state. Doubly stochastic models attribute spiking irregularity on fast timescales to a discrete point process and therefore allow the single-trial firing rate to change smoothly in time. Separating shot noise arising from discrete spikes is necessary to obtain smoothly varying firing

rates on single trials. In contrast to virtually all other methods, our approach does not bin spikes and processes data spike-by-spike in continuous time and hence does not depend on an arbitrary bin-size hyperparameter.

1.5 Population dynamics and geometry on correct and error trials

Our models fit spike data from all correct and error trials without receiving any information about animal choice (i.e., the models are not informed about which trials are correct or error). Thus, by inspecting the fitted models, we can determine whether the dynamics and geometry of neural representations were the same or different on correct versus error trials.

Our modeling framework dissociates population dynamics and geometry (Extended Data Figs. 2,3) and therefore enables us to test alternative hypotheses about how errors emerge in single-trial population activity. One plausible hypothesis is that neural trajectories take distinct paths through the population state space on correct versus error trials (Supplementary Fig. 7a), as may be suggested by differences in the corresponding trial-averaged firing rates (Supplementary Fig. 7b). In our framework, this scenario would be revealed in tuning functions being different between the left and right stimulus conditions, in which the left choice is correct versus error, respectively (and vice versa for the right choice). An alternative hypothesis is that all trajectories leading to the same choice follow the same path through the population state space irrespective of whether this choice is correct or error (Supplementary Fig. 7a), which would be revealed in tuning functions being the same for the left and right stimulus conditions. In this case, differences in trial-averaged responses (Supplementary Fig. 7b) result solely from differences in dynamics between correct and error trials. The geometry of trial-averaged trajectories (Supplementary Fig. 7b) cannot distinguish between these alternative hypotheses, because trial-averaged responses conflate the dynamics and geometry of neural representations (Extended Data Fig. 3).

Our results support the second hypothesis. We found that tuning functions were laregly the same across all stimulus conditions (Extended Data Fig. 7), indicating that neural trajectories evolve along the same manifold on correct and error trials. The dynamics on correct and error trials were distinct. On correct trials, the flow field drives the dynamics towards the correct-choice boundary (potential slope inclined towards the correct-choice boundary, Fig. 3d,g, Fig. 4c). On error trials, the dynamics first step over the potential barrier due to noise and then the flow field drives the dynamics towards the incorrect-choice boundary (potential slope inclined towards the incorrect-choice boundary). Thus, the flow field drives the dynamics in opposite directions along the manifold on correct versus error trials for the same stimulus (Supplementary Fig. 7c). Moreover, the dynamics leading to the same choice evolve faster when this choice is correct than error, as indicated by a steeper slope of the potential on the side corresponding to the correct choice (Supplementary Fig. 7c). Thus, the geometry of the choice manifold is nearly invariant to whether the choice is correct or error, whereas the speed and direction of how the dynamics evolve along this manifold differ.

These differences in dynamics between correct and error trials are nontrivial. In particular, in a drift-diffusion model, the dynamics leading to correct and error choices evolve in a linear potential with the same constant slope everywhere in the state space and differ only due to noise (Supplementary Fig. 5a). Hence, the flow field drives the dynamics towards the correct-choice boundary on both correct and error trials (Supplementary Fig. 7d). Instead, differences in dynamics on correct and error trials revealed by our model are consistent with the attractor

mechanism (Fig. 5).

2 Supplementary Methods

2.1 Analytical calculation and numerical evaluation of the likelihood

The likelihood is calculated by marginalizing the joint probability of simultaneously observing a latent trajectory $\mathcal{X}(t)$ and spike data $Y(t)$ from a model θ:

$$\mathscr{L}\left[Y(t)|\theta\right] = \int \mathscr{D}\mathcal{X}(t)\ P(\mathcal{X}(t), Y(t)|\theta). \tag{1}$$

We first integrate $P(\mathcal{X}(t), Y(t)|\theta)$ over the distribution of all latent paths in between the observed spikes to obtain the joint probability $P(X(t), Y(t)|\theta)$ where $X(t) = \{x_{t_0}, x_{t_1}, \ldots, x_{t_N}, x_{t_E}\}$ is a discretized trajectory which consists of the initial state x_{t_0}, the final state at the trial end x_{t_E}, and all states $x_{t_1}, ...x_{t_N}$ at the times of spike observations from all neurons. The likelihood is obtained by marginalizing $P(X(t), Y(t)|\theta)$ over the discretized trajectory:

$$\mathscr{L}\left[Y(t)|\theta\right] = \int_{x_{t_0}} \int_{x_{t_1}} \cdots \int_{x_{t_N}} \int_{x_{t_E}} dx_{t_0} \ldots dx_{t_E} P(X(t), Y(t)|\theta). \tag{2}$$

Using the Markov property of the latent Langevin dynamics Eq. (1) and conditional independence of spike observations, the joint probability density $P(X(t), Y(t))$ can be factorized [8]:

$$P(X(t), Y(t)) = p(x_{t_0}) \left(\prod_{i=1}^{N} p(y_{t_i}|x_{t_i}) p(x_{t_i}|x_{t_{i-1}}) \right) p(x_{t_E}|x_{t_N}) p(A|x_{t_E}). \tag{3}$$

Here $p(y_{t_i}|x_{t_i})dt$ is the probability of observing a spike from neuron k_i within a small dt of time t_i given the latent state x_{t_i}, hence $p(y_{t_i}|x_{t_i}) = f_{k_i}(x_{t_i})$ by the definition of the instantaneous Poisson firing rate, where k_i is the index of the neuron that emitted a spike at time t_i. $p(x_{t_0})$ is the probability density of the initial latent state. $p(x_{t_i}|x_{t_{i-1}})$ is the transition probability density from $x_{t_{i-1}}$ to x_{t_i} during the time interval between the adjacent spike observations from all neurons, which accounts for the absence of spikes during this time interval. This transition probability is marginalized over all intermediate latent paths connecting $x_{t_{i-1}}$ at time t_{i-1} and x_{t_i} at time t_i. Finally, the term $p(A|x_{t_E})$ is the absorption operator, which ensures that only trajectories terminating at one of the domain boundaries at time t_E contribute to the likelihood [8]. $p(x_{t_i}|x_{t_{i-1}})$ is a solution of the modified Fokker-Planck equation [8]:

$$\frac{\partial p(x,t)}{\partial t} = \left(-D \frac{\partial}{\partial x} F(x) + D \frac{\partial^2}{\partial x^2} - \sum_{k=1}^{M} f_k(x) \right) p(x,t) \equiv -\hat{\boldsymbol{\mathcal{H}}} p(x,t), \tag{4}$$

where the term $\sum_{k=1}^{M} f_k(x)$ is the total firing rate of all neurons that accounts for the absence of spikes during the interspike intervals. The absorbtion operator is $\boldsymbol{A} = \hat{\boldsymbol{\mathcal{H}}}_0$, where $\hat{\boldsymbol{\mathcal{H}}}_0$ is the Fokker-Planck operator [8]:

$$\hat{\boldsymbol{\mathcal{H}}}_0 = D \frac{\partial}{\partial x} F(x) - D \frac{\partial^2}{\partial x^2}. \tag{5}$$

To numerically calculate the likelihood using Eq. (2), we need to solve Eq. (4) for each interspike interval. To solve Eq. (4), we introduce a Hermitian operator $\boldsymbol{\mathcal{H}} = \exp(\Phi(x)/2)\hat{\boldsymbol{\mathcal{H}}}\exp(-\Phi(x)/2)$ that propagates forward in time the scaled probability density $\rho(x,t) = p(x,t)\exp(\Phi(x)/2)$:

$$\frac{\partial \rho(x,t)}{\partial t} = -\boldsymbol{\mathcal{H}}\rho(x,t). \tag{6}$$

Eq. (6) is a linear equation with a symmetric differential operator that allows for an efficient numerical solution. The operator $\boldsymbol{\mathcal{H}}$ can be decomposed into a sum of two operator: $\boldsymbol{\mathcal{H}} = \boldsymbol{\mathcal{H}}_0 + \boldsymbol{\mathcal{H}}_I$, where $\boldsymbol{\mathcal{H}}_0$ accounts for drift and diffusion in the latent space, and $\boldsymbol{\mathcal{H}}_I$ accounts for the probability decay during interspike intervals:

$$\begin{aligned} \boldsymbol{\mathcal{H}}_0 &= -e^{\Phi(x)/2}\frac{\partial}{\partial x} D e^{-\Phi(x)}\frac{\partial}{\partial x} e^{\Phi(x)/2}, \\ \boldsymbol{\mathcal{H}}_I &= \sum_{k=1}^{M} f_k(x). \end{aligned} \tag{7}$$

First, we find the eigenvalues and eigenvectors of the operator $\boldsymbol{\mathcal{H}}_0$:

$$\boldsymbol{\mathcal{H}}_0 \Psi_0(x) = \lambda \Psi_0(x). \tag{8}$$

To this end, we introduce scaled eigenfunctions $\phi_0(x) = \exp(\Phi(x)/2)\Psi_0(x)$, which are the solution of the following scaled eigenvalue problem:

$$-\frac{\partial}{\partial x} D e^{-\Phi(x)}\frac{\partial}{\partial x}\phi_0(x) = \lambda_0 e^{-\Phi(x)}\phi_0(x). \tag{9}$$

We solve the problem Eq. (9) numerically using the spectral elements method (SEM, see Supplementary Information in Refs. [8, 25] for details). We obtain the set of eigenvalues $\lambda_{0,i}$ and the eigenvectors $\phi_{0,i}(x_k)$, where $i = 1, 2, ..., N_v$ indexes the eigenvalues and eigenvectors, N_v is the number of retained eigenvalues, and k indexes grid points in the discretized domain. For all model fits, we retain all eigenvectors of the Fokker-Planck operator. The number of retained eigenvectors is $N_v = N - 2 = 447$, where $N = 449$ is the size of the SEM grid. The dimensionality of the discretized Fokker-Planck operator is $N - 2$ because the boundary conditions constrain the solution space, reducing the effective dimensionality of the problem from N degrees of freedom to $N - 2$. The problem Eq. (9) is a generalized eigenvalue problem with a non-trivial right-hand side function $\exp(-\Phi(x))$. With the SEM discretization, the mass matrix $\boldsymbol{M}$ for Eq. (9) is a product of $\exp(-\Phi(x))$ with the vector of SEM weights $\boldsymbol{w}$. The eigenvectors are orthogonal with respect to the mass matrix: ${\boldsymbol{\phi_i}}^T \boldsymbol{W}_0 \boldsymbol{\phi_j} = \delta_{ij}$, where $\boldsymbol{W}_0$ is a diagonal matrix with diagonal entries equal to the elementwise product of $\exp(-\Phi(x))$ with the vector of SEM weights $\boldsymbol{w}$.

After finding the eigenvalues $\lambda_{0,i}$ and eigenvectors $\phi_{0,i}(x)$ of the problem Eq. (9), we obtain the solution of the eigenvalue problem Eq. (8) by scaling back the eigenvectors: $\Psi_{0,i}(x) = \phi_{0,i}(x)\exp(-\Phi(x)/2)$, and the eigenvalues stay unchanged. It is convenient to represent the eigenvalues as a single vector $\boldsymbol{\lambda}_0 = \{\lambda_{0,i}\}$, and the eigenvectors as a transformation matrix that contains each eigenvector as a column $\boldsymbol{Q}_0 = \{\boldsymbol{\Psi}_0\}$. The scaling $\Psi_{0,i}(x) = \phi_{0,i}(x)\exp(-\Phi(x)/2)$ makes the new eigenvectors $\Psi(x)$ to be orthogonal with respect to the diagonal matrix of the SEM weights $\boldsymbol{W} = \mathrm{diag}(\boldsymbol{w})$, so that $\boldsymbol{Q}_0^T \boldsymbol{W} \boldsymbol{Q}_0 = \boldsymbol{I}$.

Next, we find the eigenvalues and eigenvectors of the operator $\boldsymbol{\mathcal{H}}$:

$$\boldsymbol{\mathcal{H}}\Psi(x) = \lambda\Psi(x). \tag{10}$$

To this end, we rewrite Eq. (10) in the basis of operator $\boldsymbol{\mathcal{H}}_0$. By using the definition $\boldsymbol{\mathcal{H}} = \boldsymbol{\mathcal{H}}_0 + \boldsymbol{\mathcal{H}}_I$, Eq. (10) in the basis of operator $\boldsymbol{\mathcal{H}}_0$ reads:

$$(\boldsymbol{\lambda}_0 \boldsymbol{I} + \boldsymbol{Q}_0^T \boldsymbol{W}\boldsymbol{F}\boldsymbol{Q}_0)\hat{\boldsymbol{\Psi}} = \lambda\hat{\boldsymbol{\Psi}}, \tag{11}$$

where $\boldsymbol{F}$ is the sum of tuning functions of all neurons $\sum_{k=1}^{M} f_k(x)$ discretized on the SEM grid into a diagonal matrix. After obtaining the matrix of eigenvectors $\hat{\boldsymbol{Q}}$, which is a transformation matrix from the basis of operator $\boldsymbol{\mathcal{H}}_0$ to the basis of $\boldsymbol{\mathcal{H}}$, we find the solution of the original problem as $\boldsymbol{Q} = \boldsymbol{Q}_0\hat{\boldsymbol{Q}}$. The matrix $\boldsymbol{Q}$ contains the eigenvectors $\boldsymbol{\Psi}$ that solve the problem Eq. (10). The eigenvalues of Eq. (10) are the same as for Eq. (11), since the eigenvalues are basis independent.

In summary, to find the time-dependent solution of Eq. (6), we solve the corresponding eigenvalue problem Eq. (10). This problem is solved by finding the first N_v eigenvalues and eigenvectors of Eq. (9), then multiplying the eigenfunctions ϕ_0 by $\exp(-\Phi(x)/2)$ to obtain the eigenfunctions Ψ_0 of the problem Eq. (8) that constitute the matrix $\boldsymbol{Q}_0$. Using these eigenvectors and the eigenvalues $\boldsymbol{\lambda}_0$, we then solve another eigenvalue problem Eq. (11), from which we obtain the eigenvalues $\boldsymbol{\lambda}$ and the eigenvectors $\hat{\boldsymbol{Q}}$. The eigenvalues $\boldsymbol{\lambda}$ and the eigenvectors $\boldsymbol{Q} = \boldsymbol{Q}_0\hat{\boldsymbol{Q}}$ are then the solution of Eq. (10).

For numerical solution, we discretize all functions and the eigenvalue problems in the SEM grid [8]. Thus, all probability densities in Eq. (3) become vectors of size N and all operators become matrices of size N^2, where N is the number of grid points (we set $N = 449$ for all model fits). We use a Gauss–Legendre–Lobatto (GLL) grid with Lagrange interpolating polynomials as basis functions (see Methods and Supplementary Information in Refs. [8, 25] for details). The scaled transition probability density $\rho(x_{t_i}|x_{t_{i-1}})$ is solution of Eq. (6) and can be expressed in the basis of operator $\boldsymbol{\mathcal{H}}$ as:

$$\boldsymbol{\rho}_{i,i-1} \equiv \boldsymbol{T}_i = \operatorname{diag}\left(\exp\left(-\boldsymbol{\lambda}(t_i - t_{i-1})\right)\right), \tag{12}$$

where $\boldsymbol{\rho}_{i,i-1}$ is the transition matrix over latent states between the times t_{i-1} and t_i of the adjacent spikes. When the scaled probability density of latent states $\boldsymbol{\rho}_j$ at time t_j is multiplied by the transition matrix $\boldsymbol{\rho}_{j+1,j}$ between the times t_j and t_{j+1}, the result is the scaled probability density $\boldsymbol{\rho}_{j+1}$ at time t_{j+1}, since the matrix-vector product marginalizes over the distribution of latent states at time t_j. Similarly, the emission matrix, which gives the probability of observing a spike from neuron k conditioned on the latent state, is expressed in the basis of operator $\boldsymbol{\mathcal{H}}$ as:

$$\boldsymbol{E}_k = \boldsymbol{Q}^T\boldsymbol{W}\operatorname{diag}(\boldsymbol{f}_{\mathrm{k}})\boldsymbol{Q}, \tag{13}$$

where $\boldsymbol{f}_k$ is a vector of the discretized tuning function $f_k(x)$. The matrix of the absorption operator $\boldsymbol{A} = \hat{\boldsymbol{\mathcal{H}}}_0$ in the $\boldsymbol{\mathcal{H}}$-basis is:

$$\boldsymbol{A} = \hat{\boldsymbol{Q}}^T\operatorname{diag}(\boldsymbol{\lambda}_0)\hat{\boldsymbol{Q}}, \tag{14}$$

where $\hat{\boldsymbol{Q}}$ are the eigenvectors of the problem Eq. (11).

We can rewrite Eqs. (2), (3) in the finite basis of operator $\boldsymbol{\mathcal{H}}$ to obtain the chain of vector-matrix multiplications:

$$\mathscr{L} = \boldsymbol{\rho}_0^T\boldsymbol{T}_1\boldsymbol{E}_{k_1}\boldsymbol{T}_2\boldsymbol{E}_{k_2}\cdots\boldsymbol{T}_{N+1}\boldsymbol{A}\boldsymbol{\beta}_{N+2}. \tag{15}$$

Here $\boldsymbol{\beta}_{N+2}$ is a column vector used to integrate the likelihood over the final state x_{t_E}. This vector is equal to $\boldsymbol{\beta}_{N+2} = \boldsymbol{\rho}_{\text{eq}} \boldsymbol{w}$, where $\boldsymbol{\rho}_{\text{eq}}$ is a discretized vector of $\rho_{\text{eq}}(x) = \sqrt{p_{\text{eq}}} = \exp(-\Phi(x)/2)$ that scales $\rho(x,t)$ back into $p(x,t)$ and sums it up with the vector of SEM weights to integrate over x_{t_E}. In Eq. (15), the indices $k_1, k_2, \dots k_N$ refer to the index of a neuron emitting a spike at times $t_1, t_2, \dots t_N$, and the spike times are ordered across all neurons.

We evaluate Eq. (15) with a forward pass that calculates the chain from left to right:

$$
\begin{aligned}
&\boldsymbol{\alpha_0}^T = \boldsymbol{\rho_0}^T, \qquad \boldsymbol{\alpha_n}^T = \boldsymbol{\alpha_{n-1}}^T \boldsymbol{T}_n \boldsymbol{E}_{k_n},\ n = 1, 2 \dots N, \\
&\boldsymbol{\alpha_{N+1}}^T = \boldsymbol{\alpha_N}^T \boldsymbol{T}_{N+1}, \qquad \boldsymbol{\alpha_{N+2}}^T = \boldsymbol{\alpha_{N+1}}^T \boldsymbol{A},
\end{aligned}
\tag{16}
$$

with $\mathscr{L} = \boldsymbol{\alpha_{N+2}}^T \boldsymbol{\beta_{N+2}}$. We use a scaling algorithm similar to that for the Hidden Markov Models [30]. After calculating each $\boldsymbol{\alpha_n}$, we calculate $c_n = ||\boldsymbol{\alpha_n}||$ and divide each $\boldsymbol{\alpha_n}$ by its norm before calculating $\boldsymbol{\alpha_{n+1}}$. With the scaling algorithm, each $\boldsymbol{\alpha_n}$ has the norm equal to 1, and the likelihood is equal to the product of all coefficients c, that is, $\log \mathscr{L} = \sum \log c_n$.

To compare the likelihood between the single-neuron and population models and between the single-neuron model and trial-averaged firing rate (PSTH), we normalize the full model likelihood by the likelihood $\mathscr{L}_{t_E}$ of the latent trajectory reaching a boundary for the first time at time t_E. This latter likelihood is computed via:

$$
\mathscr{L}_{t_E} = \boldsymbol{\alpha_0}^T \hat{\boldsymbol{T}}_E \boldsymbol{A} \boldsymbol{\beta_{N+2}},
\tag{17}
$$

where $\hat{\boldsymbol{T}}_E$ is the matrix propagating the probability density of the latent state from trial start t_0 to the trial end time $t = t_E$ that only accounts for latent trajectory dynamics and ignores spike observations. This propagation matrix is computed from the solution of eigenvalue-eigenvector problem for the operator $\boldsymbol{\mathcal{H}}_0$ (Eq. 8). In the basis of $\boldsymbol{\mathcal{H}}_0$ operator, it is computed as:

$$
\hat{\boldsymbol{T}}_E = \operatorname{diag}\left(\exp\left(-\boldsymbol{\lambda}_0 (t_E - t_0)\right)\right).
\tag{18}
$$

2.2 Analytical derivation of the likelihood derivatives

We have previously derived the expressions for the variational derivatives of the likelihood with respect to the force $F(x)$, the auxiliary function $F_0(x)$, and the noise magnitude D [8]:

$$
\begin{aligned}
\frac{\delta \mathscr{L}}{\delta F(x)} &= \sum_{ij} G_{ij} \frac{D}{2} e^{-\Phi(x)} \frac{d\left(\phi_i(x)\phi_j(x)\right)}{dx} + \frac{1}{2}\int_{-1}^{x} \left(\beta_0(s) p_0(s) e^{\Phi(s)/2} - \alpha_{N+2}(s) e^{-\Phi(s)/2}\right) ds, \\
\frac{\delta \mathscr{L}}{\delta F_0(x)} &= \int_{-1}^{x} p_0(s) \left(\mathscr{L} - e^{\Phi(s)/2}\beta_0(s)\right) ds, \\
\frac{\partial \mathscr{L}}{\partial D} &= -\int_{-1}^{1} dx e^{-\Phi(x)} \sum_{ij} G_{ij} \frac{d\phi_i(x)}{dx}\frac{d\phi_j(x)}{dx}.
\end{aligned}
\tag{19}
$$

Here the matrix $\boldsymbol{G}$ is computed with a forward-backward algorithm [30]. First, we perform the forward pass using Eq. (16). Then, we perform the backward path via a series of matrix-vector multiplications:

$$
\begin{aligned}
&\boldsymbol{\beta}_{N+1} = \boldsymbol{A}\boldsymbol{\beta}_{N+2}, \\
&\boldsymbol{\beta}_n = \boldsymbol{E}_{k_{N+1}} \boldsymbol{T}_{N+1} \boldsymbol{\beta}_{n+1}, \qquad n = 1, 2 \dots N, \\
&\boldsymbol{\beta}_0 = \boldsymbol{T}_1 \boldsymbol{\beta}_1.
\end{aligned}
\tag{20}
$$

We compute the matrix $\boldsymbol{G}$ during the backward pass:

$$G_{ij} = \sum_{\tau=0}^{N+1} \Gamma_{ij}^{\tau+1} \alpha_{\tau,i} \beta_{\tau+1,j}, \tag{21}$$

where the matrix Γ_{ij}^{τ} is equal to the negative identity matrix for $\tau = N+2$, $\boldsymbol{\Gamma}^{N+2} = -\boldsymbol{I}$, and otherwise:

$$\Gamma_{ij}^{\tau} = \int_0^{\Delta t_\tau} e^{-(\Delta t_\tau - u)\lambda_i} e^{-u\lambda_j} du = \begin{cases} \Delta t_\tau e^{-\lambda_i \Delta t_\tau}, & i = j, \\ \frac{e^{-\lambda_i \Delta t_\tau} - e^{-\lambda_j \Delta t_\tau}}{\lambda_j - \lambda_i}, & i \neq j, \end{cases} \tag{22}$$

where $\Delta t_\tau = t_\tau - t_{\tau-1}$.

In this work, we also perform the inference of tuning functions $f_k(x)$ via the auxiliary functions $F_k(x)$ and auxiliary variables C_k. To derive the analytical expressions for these derivatives, we follow similar steps as in Ref. [8]. We write the expression for the likelihood in terms of propagation and emission operators:

$$\mathscr{L}\left[Y(t)|\theta\right] = \left\langle \rho_0 \middle| e^{-\boldsymbol{\mathcal{H}}\Delta t_1} \boldsymbol{y}_{k_1} e^{-\boldsymbol{\mathcal{H}}\Delta t_2} \boldsymbol{y}_{k_2} ... e^{-\boldsymbol{\mathcal{H}}\Delta t_N} \boldsymbol{y}_{k_N} e^{-\hat{\boldsymbol{\mathcal{H}}}\Delta t_{N+1}} \boldsymbol{A} \middle| \beta_{N+2} \right\rangle, \tag{23}$$

where we use the bra-ket notation for the state vectors and operator matrices. This form is basis independent and allows for the analytical likelihood calculation. In this notation, row vectors, such as $\boldsymbol{\rho}_0^T$, correspond to bra states $\langle \rho_0|$, propagation matrices, such as $\boldsymbol{T}_i$,correspond to the exponential operators $\exp\left(-\boldsymbol{\mathcal{H}}(t_i - t_{i-1})\right)$, spike emission matrices correspond to the spike emission operators, and column vectors, such as $\boldsymbol{\beta}_{N+2}$, correspond to ket states $|\beta_{N+2}\rangle$.

First, we compute the derivative of the likelihood with respect to the tuning function $f_k(x)$ of neuron k. In Eq. (23), each of the propagation operators depends on $f_k(x)$. In addition, the emission operators for which $k_i = k$ also depend on $f_k(x)$. Using the formula for product of the derivatives, we obtain:

$$\frac{\delta \mathscr{L}}{\delta f_k(x)} = \sum_{i,j} \left[\sum_{\tau=1}^{N+1} a_i(\tau-1) b_j(\tau) \frac{\delta \langle \Psi_i | e^{-\boldsymbol{\mathcal{H}}\Delta t_\tau} | \Psi_j \rangle}{\delta f_k(x)} + \sum_{\tau_2} \hat{a}_i(\tau_2 - 1) \hat{b}_j(\tau_2) \frac{\delta \langle \Psi_i | f_k(x) | \Psi_j \rangle}{\delta f_k(x)} \right], \tag{24}$$

where in the second sum, the index τ_2 takes the values of indices corresponding to spikes of the neuron k. The quantities $a, b, \hat{a}, \hat{b}$ are obtained via two forward and backward passes:

$$\begin{aligned}
&\langle \alpha_0 | = \langle \rho_0 |, \qquad \langle \alpha_n | = \langle \alpha_{n-1} | e^{-\boldsymbol{\mathcal{H}}\Delta t_n} \boldsymbol{y}_{k_n},\ n = 1, 2 \ldots N, \\
&\langle \alpha_{N+1} | = \langle \alpha_N | e^{-\boldsymbol{\mathcal{H}}\Delta t_{N+1}}, \qquad \langle \alpha_{N+2} | = \langle \alpha_{N+1} | \boldsymbol{A}, \\
&|\beta_{N+1}\rangle = \boldsymbol{A} |\beta_{N+2}\rangle, \\
&|\beta_n\rangle = \boldsymbol{y}_{k_n} e^{-\boldsymbol{\mathcal{H}}\Delta t_{n+1}} |\beta_{n+1}\rangle,\ n = 1, 2, \ldots N, \qquad |\beta_0\rangle = e^{-\boldsymbol{\mathcal{H}}\Delta t_1} |\beta_1\rangle, \\
&a_i(\tau) = \langle \alpha_\tau | \Psi_i \rangle, \qquad b_j(\tau) = \langle \Psi_j | \beta_\tau \rangle.
\end{aligned} \tag{25}$$

$$\begin{aligned}
&\langle \hat{\alpha_0} | = \langle \rho_0 | e^{-\boldsymbol{\mathcal{H}}\Delta t_1}, \qquad \langle \hat{\alpha}_n | = \langle \hat{\alpha}_{n-1} | \boldsymbol{y}_{k_n} e^{-\boldsymbol{\mathcal{H}}\Delta t_{n+1}},\ n = 1, 2 \ldots N, \\
&|\hat{\beta}_N\rangle = e^{-\boldsymbol{\mathcal{H}}\Delta t_{N+1}} \boldsymbol{A} |\hat{\beta}_{N+2}\rangle, \qquad |\hat{\beta}_n\rangle = e^{-\boldsymbol{\mathcal{H}}\Delta t_{n+1}} \boldsymbol{y}_{k_n+1} |\hat{\beta}_{n+1}\rangle, \\
&\hat{a}_i(\tau) = \langle \hat{\alpha}_\tau | \Psi_i \rangle, \qquad \hat{b}_j(\tau) = \langle \Psi_j | \hat{\beta}_\tau \rangle.
\end{aligned} \tag{26}$$

In the discretized space, $\langle\alpha_\tau|$ correspond to the forward vectors $\boldsymbol{\alpha}_\tau$ in Eq. (16), and $|\beta_\tau\rangle$ correspond to the backward vectors $\boldsymbol{\beta}_\tau$ in Eq. (20) with components $\alpha_i(\tau)$ and $\beta_i(\tau)$, respectively.

Using the formula for the derivative of the exponential operator, we obtain[31]:

$$\frac{\delta\langle\Psi_i|e^{-\boldsymbol{\mathcal{H}}\Delta t_\tau}|\Psi_j\rangle}{\delta f_k(x)} = -\left\langle\Psi_i\left|\frac{\delta\boldsymbol{\mathcal{H}}}{\delta f_k(x)}\right|\Psi_j\right\rangle\Gamma^\tau_{i,j}, \tag{27}$$

where the matrix Γ is defined in Eq. (22). Operator $\boldsymbol{\mathcal{H}}$ depends on $f_k(x)$ through the term $\boldsymbol{\mathcal{H}}_I = \sum_{k=1}^M f_k(x)$, and we compute its variational derivative using the Euler-Lagrange equation:

$$\left\langle\Psi_i\left|\frac{\partial\boldsymbol{\mathcal{H}}}{\partial f_k(x)}\right|\Psi_j\right\rangle = \Psi_i(x)\Psi_j(x). \tag{28}$$

As a result, we obtain for the first term on the right hand side of Eq. (24):

$$\sum_{\tau=1}^{N+1} a_i(\tau-1)b_j(\tau)\frac{\delta\langle\Psi_i|e^{-\boldsymbol{\mathcal{H}}\Delta t_\tau}|\Psi_j\rangle}{\delta fr_k} = -\Psi_i(x)\Psi_j(x)\hat{G}_{ij}, \tag{29}$$

where

$$\hat{G}_{ij} = \sum_{\tau=0}^{N}\Gamma^{\tau+1}_{ij}\alpha_{\tau,i}\beta_{\tau+1,j}. \tag{30}$$

Note that $\hat{G}_{ij}$ is the same as G_{ij} defined in Eq. (21) except for the last term, since the absorption operator does not depend on the tuning function and hence does not contribute to the derivative. We similarly evaluate the second term on the right hand side of Eq. (24) and obtain the likelihood derivative with respect to the tuning function:

$$\frac{\delta\mathscr{L}}{\delta f_k(x)} = \sum_{ij}\Psi_i(x)\Psi_j(x)(G^{(k)}_{ij} - \hat{G}_{ij}), \tag{31}$$

with the matrix $G^{(k)}_{ij}$ is defined as:

$$G^{(k)}_{ij} = \sum_{\tau_2}\hat{\alpha}_{\tau_2,i}\hat{\beta}_{\tau_2+1,j}, \tag{32}$$

where τ_2 runs through all time points where neuron k spiked.

Next, we compute the likelihood derivatives with respect to the auxiliary function $F_k(x)$ and the auxiliary variable C_k used in the optimization. We note that

$$\frac{\partial f_k(x)}{\partial C_k} = \frac{f_k(x)}{C_k}. \tag{33}$$

To evaluate the derivative of $f_k(x)$ with respect to $F_k(x)$, we introduce another auxiliary function $R_k(x) = \int_{-1}^1 F_k(x')H(x-x')dx' = \int_{-1}^x F_k(x')dx'$, where $H(s)$ is the Heaviside step function. With this substitution, $f_k(x) = C_k\exp(R_k(x))$, hence:

$$\begin{aligned}\frac{\delta R_k(s')}{\delta F_k(s)} &= H(s'-s),\\ \frac{\delta f_k(x)}{\delta R_k(s')} &= C_k e^{R_k(x)}\delta(s'-x).\end{aligned} \tag{34}$$

Using this results, we obtain:

$$\frac{\delta f_k(x)}{\delta F_k(s)} = \int_{-1}^{1} \frac{\delta f_k(x)}{\delta R_k(s')} \frac{\delta R_k(s')}{\delta F_k(s)} ds' = C_k e^{R_k(x)} \int_{-1}^{1} \delta(s'-x) H(s'-s) ds' = f_k(x) H(x-s). \tag{35}$$

Using the results in Eqs. (31), (35), we obtain the final expressions:

$$\begin{aligned} \frac{\delta \mathscr{L}}{\delta F_k(x)} = \int_{-1}^{1} \frac{\delta \mathscr{L}}{\delta f_k(s)} \frac{\delta f_k(s)}{\delta F_k(x)} ds = \int_{-1}^{1} \frac{\delta \mathscr{L}}{\delta f_k(s)} f_k(s) H(s-x) ds = \\ = \int_{-1}^{1} \frac{\delta \mathscr{L}}{\delta f_k(s)} f_k(s) ds - \int_{-1}^{x} \frac{\delta \mathscr{L}}{\delta f_k(s)} f_k(s) ds, \end{aligned} \tag{36}$$

and for the derivative with respect to the constants C_k we obtain:

$$\frac{\partial \mathscr{L}}{\partial C_k} = \int_{-1}^{1} \frac{\delta \mathscr{L}}{\delta f_k(s)} \frac{\partial f_k(s)}{\partial C_k} ds = \frac{1}{C_k} \int_{-1}^{1} \frac{\delta \mathscr{L}}{\delta f_k(s)} f_k(s) ds. \tag{37}$$

In summary, we compute the derivatives of the likelihood with respect to F, F_0 and D using Eq. (19) derived in Ref. [8]. The derivatives with respect to $F_k(x)$ and C_k for each neuron k are computed using Eqs. (36), (37) and (31).

2.3 Maximum likelihood optimization with ADAM algorithm

We optimized the model likelihood using a modified ADAM gradient-descent algorithm combined with line searches. The ADAM update on iteration t is:

$$\begin{aligned} m_t &= \beta_1 m_{t-1} + (1-\beta_1) g_t, \\ \hat{m}_t &= m_t / (1-\beta_1^t), \\ v_t &= \beta_2 v_{t-1} + (1-\beta_2) ||g_t||^2, \\ \hat{v}_t &= v_t/(1-\beta_2^t), \\ \theta_t &= \theta_{t-1} - \alpha \frac{\hat{m}_t}{\sqrt{\hat{v}_t} + \epsilon}, \end{aligned} \tag{38}$$

where α, β_1, β_2, ϵ are hyperparameters, m_t is a running average of the gradient g_t on iteration t, v_t is a running average of the gradient's L^2 squared norm, $\hat{m}_t$ and $\hat{v}_t$ are computed from m_t and v_t to correct the initialization bias (at zero iteration $m_0 = v_0 = 0$). We apply Eqs. (38) independently to each of the model's components $F(x), F_0(x), F_i(x), D, C_i$. The main difference between the original ADAM algorithm and our version is that we optimize over the space of continuous functions $F(x)$, $F_0(x)$, and $\{F_i(x)\}$ and not independent discrete parameters, therefore we scale their gradients by the average function's L^2-norm defined as $\|\phi(x)\|_2 = \int_x |\phi(x)|^2 dx$.

On each epoch of ADAM, we randomly group the trials into 20 identical trial batches (with the exception of the last batch, which may contain less trials if the total number of trials is not divisible by 20). Each epoch thus consisted of 20 iterations. We then compute ADAM quantities and update each of the model components using Eqs. (38) on each batch of trials. In addition, on a subset of epochs, we performed line searches for the scalar parameters D and each C_i using L-BFGS-B method from scipy.optimize.minimize toolbox. Since a line search is computationally

expensive, we perform only 30 line searches spaced logarithmically over the 5,000 epochs range, such that most line searches are concentrated at early epochs.

To perform shared optimization across four stimulus conditions, we randomly split trials for each of the four conditions into 20 batches of equal size, resulting in 80 batches total. We randomly permuted the order of these 80 batches, but all trials in one batch come from the same condition. For each trial batch, we calculated the gradients and updated the model components Eqs. 38. In this case, we optimized four potential functions $\Phi_1(x), \Phi_2(x), \Phi_3(x), \Phi_4(x)$, each of which was updated only on trial batches from the corresponding condition. Other parameters, including D, $F_i(x)$, C_i and $F_0(x)$ were the same for all conditions, and thus were updated 80 times per epoch on all trial batches.

For bootstrapping, we first randomly divided our dataset into two equally sized data samples, and then sampled the trials randomly with replacement from each of the data samples. Thus, our bootstrap samples from two data halves did not intersect with each other but both of them contained repeated trials.

2.4 Model selection

We used the model selection procedure based on feature consistency that we developed in Ref. [8]. First, we introduce feature complexity of a model $\mathcal{M} = -S[\Phi(x), D, p_0(x); \Phi^R(x), D^R, p_0^R(x)]$ defined as a negative trajectory entropy. The trajectory entropy is defined as a negative Kullback-Leibler (KL) divergence between the distributions $P[\mathcal{X}(t)]$ and $Q[\mathcal{X}(t)]$ [32]:

$$S[\Phi(x), D, p_0(x); \Phi^R(x), D^R, p_0^R(x)] = -\int_0^{t_{\text{obs}}} \mathscr{D}\mathcal{X}(t) P\left[\mathcal{X}(t)\right] \ln \frac{P\left[\mathcal{X}(t)\right]}{Q\left[\mathcal{X}(t)\right]}. \tag{39}$$

$P[\mathcal{X}(t)]$ is the distribution of trajectories in the model of interest with Langevin parameters $\{\Phi(x), D, p_0(x)\}$, and $Q[\mathcal{X}(t)]$ is the distribution of trajectories in the reference model with Langevin parameters $\{\Phi^R(x), D^R, p_0^R(x)\}$. The path integral is performed over all possible trajectories $\mathcal{X}(t)$. The reference model is a free diffusion with zero driving force (i.e. constant potential $\Phi^R(x) = \text{const}$), uniform $p_0^R(x)$, and the same diffusion coefficient D as in the model of interest. Intuitively, feature complexity quantifies the number and prominence of features in the model: the greater $\mathcal{M}$ the more complex trajectories the model generates.

We previously derived an analytical expression for the trajectory entropy through the Langevin parameters [8]:

$$S[\Phi(x), D, p_0(x); \Phi^R(x), D, p_0^R(x)] = -\int_x dx p_0(x) \ln \frac{p_0(x)}{p_0^R(x)} - \frac{D}{4} \int_0^\infty dt \int_x dx F^2(x) p(x,t). \tag{40}$$

To evaluate Eq. (40) numerically, we integrate the first term using the Gaussian quadrature on a Gauss-Legendre-Lobatto (GLL) grid:

$$\int dx p_0(x) \ln \frac{p_0(x)}{p_0^R(x)} = \sum_k p_0(x_k) \ln \frac{p_0(x_k)}{p_0^R(x_k)} w_k, \tag{41}$$

where w_k are the GLL integration weights. The second term can be simplified by switching into the basis of operator $\boldsymbol{\mathcal{H}}$ and evaluating the integral over time analytically [8]:

$$\int_0^\infty dt \int dx F^2(x) p(x,t) = \sum_k \frac{\rho_{0,k} (F^2 \exp[-\Phi/2])_k}{\lambda_k}. \tag{42}$$

Here k indexes the elements of the vectors in the basis of operator $\boldsymbol{\mathcal{H}}$, $\rho_0(x) = p_0(x)/\sqrt{p_{\text{eq}}(x)}$, and λ_k are the eigenvalues of operator $\boldsymbol{\mathcal{H}}$. Thus, Eqs. (40–42) allow us to compute feature complexity from the Langevin parameters $\Phi(x)$, $p_0(x)$, and D.

We split our full dataset into two halves $\mathcal{D}_1$ and $\mathcal{D}_2$ and optimize the model on each data split independently. As a result, we obtain two sequences of models $\theta_{1,n}$ and $\theta_{2,n}$, where $n = 1, 2, \ldots 5,000$ is the epoch number. To reduce the amount of computations needed for model selection, we then subsample these models on a logarithmic scale by choosing 400 models indexed by m where $m \propto \log n$. For each of the sub sampled models we then calculate feature complexity $\mathcal{M}(\Phi(x), p_0(x), D)$ and obtain two sequences $\mathcal{M}_{1,m}$, and $\mathcal{M}_{2,m}$ for $m = 1, 2, ..., 400$. For each level of feature complexity, we compare two models optimized on $\mathcal{D}_1$ and $\mathcal{D}_2$. We quantify the consistency of features between models using Jansen-Shannon divergence between their time-dependent probability distributions [8]:

$$D_{\text{JS}} = \int_0^\infty \text{JSD}\left(\hat{p}^1(x,t)||\hat{p}^2(x,t)\right) dt. \tag{43}$$

Here $\hat{p}^1(x,t)$ and $\hat{p}^2(x,t)$ are the time-dependent probability densities of latent states generated by the two models. The Jason-Shanon divergence between two distributions is computed as:

$$\begin{aligned} \text{JSD}(\hat{p}^1(x)||\hat{p}^2(x)) = \frac{1}{2}\left(\int \hat{p}^1(x)\log\frac{2\hat{p}^1(x)}{\hat{p}^1(x)+\hat{p}^2(x)}dx + \int \hat{p}^2(x)\log\frac{2\hat{p}^2(x)}{\hat{p}^1(x)+\hat{p}^2(x)}dx\right) + \\ + \frac{1}{2}\left(I_1 \log\frac{2I_1}{I_1+I_2} + I_2\log\frac{2I_2}{I_1+I_2}\right), \end{aligned} \tag{44}$$

where $I_{1,2} = 1 - \int \hat{p}^{1,2}(x)dx$. We compute D_{JS} by a forward Euler time discretization of Eq. (43), where for each time step the integral Eq. (44) is computed with the GLL integration weights w_k.

At early optimization epochs the models of similar feature complexities optimized on two different datasamples agree with each other. At some feature complexity level $\mathcal{M}^*$ the two models start to disagree due to overfitting to noise which produces features that are different for two independent data samples. We numerically identify $\mathcal{M}^*$ at the point where D_{JS} exceeds a threshold $D_{\text{thres}} = 0.0015$.

Since feature complexities do not match exactly between the two model sets due to nuances in the data, we need to allow for some slack in feature complexity when comparing models [8]. Accordingly, for an iteration m_1 on the data split $\mathcal{D}_1$, we first find iteration $\hat{m}_2$ on $\mathcal{D}_2$ which minimizes $|\mathcal{M}_{1,m_1} - \mathcal{M}_{2,\hat{m}_2}|$. Next, we swipe $m_2 = \hat{m}_2 - 5, \hat{m}_2 - 4, \ldots \hat{m}_2 + 5$, and select index m_2 that minimizes D_{JS} between models m_1 and m_2, and set $D_{\text{JS}}(\mathcal{M}_{1,m_1})$ to this minimum value. We repeat this procedure for different epochs to obtain the dependence $D_{\text{JS}}(\mathcal{M})$ to which we apply the threshold $D_{\text{JS,thres}}$. To reduce the computational cost, we evaluate $D_{\text{JS}}(\mathcal{M})$ on a subset of 400 epochs spaced logarithmically over the 5,000 optimization epochs range, so that earlier epochs are sampled more densely. Before thresholding, we smooth $D_{\text{JS,thres}}$ with a running average filter of width 10.

2.5 Viterbi algorithm

We generalized the max-sum Viterbi algorithm with backtracking [30] to our case of continuous-space continuous-time latent dynamical system. The goal of the Viterbi algorithm is to find a

path $X(t)$ that maximizes the joint probability density $P(X(t), Y(t))$ in a particular trial. The discretized path $X(t) = \{x_{t_0}, x_{t_1}, \ldots, x_{t_N}, x_{t_E}\}$ is the sequence of states at the trial start time, each of the spike times, and the trial end time. We describe the Viterbi algorithm for a single-neuron model, and the extension to a population model is straightforward.

We maximize the logarithm of the joint probability density:

$$\log P(X(t), Y(t)) = \log p(x_{t_0}) + \log p(x_{t_1}|x_{t_0}) + \log p(y|x_{t_1}) + \log p(x_{t_2}|x_{t_1}) \cdots + \log p(A|x_{t_E}). \tag{45}$$

In Eq (45), only the first two terms depend on x_{t_0}, and their sum is the joint probability density $\log p(x_{t_1}, x_{t_0})$. To compute the joint probability density in the discretized latent domain with N grid points, we transform the vector $\log p(x_{t_0})$ into an $N \times N$ matrix with the same rows and sum it with the $N \times N$ matrix representing $\log p(x_{t_1}|x_{t_0})$. This operation is similar to the likelihood computation but without integration over x_{t_0}. We then take the maximum over x_{t_0} to obtain a vector $A_1(x_{t_1})$:

$$A_1(x_{t_1}) = \max_{x_{t_0}} \{\log p(x_{t_0}) + \log p(x_{t_1}|x_{t_0})\}. \tag{46}$$

In addition, we compute another vector $B_1(x_{t_1})$ that for each x_{t_1} returns the index of x_{t_0} (1 to N) that maximizes this expression:

$$B_1(x_{t_1}) = \operatorname*{argmax}_{x_{t_0}} \{\log p(x_{t_0}) + \log p(x_{t_1}|x_{t_0})\}. \tag{47}$$

Similarly, we continue evaluating the terms in Eq. (45): we take the vector $A_1(x_{t_1})$, transform it into $N \times N$ matrix, add it to the terms that depend on x_{t_1}: $\log p(y|x_{t_1}) + \log p(x_{t_2}|x_{t_1})$, and compute the vectors $A_2(x_{t_2})$ and $B_2(x_{t_2})$ by taking the maximum and argmaximum over x_{t_1}, and so on. In the end of this procedure, we have computed the whole chain and we find the maximized joint probability density:

$$\max_{X(t)} [\log P(X(t), Y(t))] = \max_{x_{t_E}} [A_{N+1}(x_{t_E}) + \log p(A|x_{t_E})]. \tag{48}$$

To evaluate the most probable sequence of latent states, we then perform backtracking. Using the value of $x^*_{t_E}$ that maximizes Eq. (48), we find the latent state at the last spike time by retrieving its index: $x^*_{t_N} = x[B_{N+1}(x^*_{t_E})]$, and then continue backtracking until we recover the whole sequence of states that maximizes $P(X(t), Y(t))$.

For our case of absorbing boundary conditions, we introduce an additional modification to the Viterbi algorithm forcing the latent trajectory to reach one of the boundaries for the first time exactly at the trial end time. First, after adding each of the latent propagation terms, we zero out all boundary elements in the $N \times N$ matrix before the max operation Eq. (46), which prevents the latent trajectory reaching a boundary before the trial end. Second, on the last step Eq. (48), we restrict $x^*_{t_E}$ to be either the left or right boundary, $x^*_{t_E} = -1$ or $x^*_{t_E} = 1$, depending on which of the two values maximizes $\log P(X(t), Y(t))$ in Eq. (48).

3 Supplementary References

[1] Kriegeskorte, N. & Wei, X.-X. Neural tuning and representational geometry. *Nat. Rev. Neurosci.* **22**, 703–718 (2021).

[2] Churchland, M. M. & Shenoy, K. V. Temporal complexity and heterogeneity of single-neuron activity in premotor and motor cortex. *Journal of Neurophysiology* **97** (2007).

[3] Churchland, M. M., Cunningham, J. P., Kaufman, M. T., Ryu, S. I. & Shenoy, K. V. Cortical Preparatory Activity: Representation ofÂ Movement or First Cog in a Dynamical Machine? *Neuron* **68**, 387–400 (2010).

[4] Mante, V., Sussillo, D., Shenoy, K. V. & Newsome, W. T. Context-dependent computation by recurrent dynamics in prefrontal cortex. *Nature* **503**, 78–84 (2013).

[5] Langdon, C., Genkin, M. & Engel, T. A. A unifying perspective on neural manifolds and circuits for cognition. *Nat. Rev. Neurosci.* **24**, 363–377 (2023).

[6] Svoboda, K. & Inagaki, H. Discrete attractor dynamics underlies persistent activity in the frontal cortex. *Figshare* (2019). Dataset at https://doi.org/10.25378/janelia.7489253.v3.

[7] Inagaki, H. K., Fontolan, L., Romani, S. & Svoboda, K. Discrete attractor dynamics underlies persistent activity in the frontal cortex. *Nature* **566**, 212 – 217 (2019).

[8] Genkin, M., Hughes, O. & Engel, T. A. Learning non-stationary Langevin dynamics from stochastic observations of latent trajectories. *Nat. Commun.* **12**, 5986 (2021).

[9] Galgali, A. R., Sahani, M. & Mante, V. Residual dynamics resolves recurrent contributions to neural computation. *Nat. Neurosci.* **26**, 326–338 (2023).

[10] O'Shea, D. J. *et al.* Direct neural perturbations reveal a dynamical mechanism for robust computation. bioRxiv preprint at https://www.biorxiv.org/content/10.1101/2022.12.16.520768v1 (2022).

[11] Wang, X. J. Probabilistic decision making by slow reverberation in cortical circuits. *Neuron* **36**, 955 – 968 (2002).

[12] Wong, K.-F. & Wang, X.-J. A recurrent network mechanism of time integration in perceptual decisions. *J. Neurosci.* **26**, 1314–1328 (2006).

[13] Jazayeri, M. & Ostojic, S. Interpreting neural computations by examining intrinsic and embedding dimensionality of neural activity. *Curr. Opin. Neurobiol.* **70**, 113–120 (2021).

[14] Pandarinath, C. *et al.* Inferring single-trial neural population dynamics using sequential auto-encoders. *Nat. Methods* **15**, 805–815 (2018).

[15] Keshtkaran, M. R. *et al.* A large-scale neural network training framework for generalized estimation of single-trial population dynamics. *Nat. Methods* **19**, 1572–1577 (2022).

[16] Hurwitz, C. *et al.* Targeted neural dynamical modeling. In *Advances in Neural Information Processing Systems* (2021).

[17] Cohen, Z., DePasquale, B., Aoi, M. C. & Pillow, J. W. Recurrent dynamics of prefrontal cortex during context-dependent decision-making. bioRxiv preprint at https://www.biorxiv.org/content/10.1101/2020.11.27.401539v1 (2020).

[18] Schimel, M., Kao, T.-C., Jensen, K. T. & Hennequin, G. iLQR-VAE : Control-based learning of input-driven dynamics with applications to neural data. In *International Conference on Learning Representations* (2022).

[19] Soldado-Magraner, J., Mante, V. & Sahani, M. Inferring context-dependent computations through linear approximations of prefrontal cortex dynamics. *Science Advances* **10**, eadl4743 (2024).

[20] Engel, T. A. *et al.* Selective modulation of cortical state during spatial attention. *Science* **354**, 1140–1144 (2016).

[21] Recanatesi, S., Pereira-Obilinovic, U., Murakami, M., Mainen, Z. & Mazzucato, L. Metastable attractors explain the variable timing of stable behavioral action sequences. *Neuron* **110**, 139–153.e9 (2022).

[22] Zhao, Y. & Park, I. M. Variational online learning of neural dynamics. *Front. Comput. Neurosci.* **14**, article 71 (2020).

[23] Duncker, L., Bohner, G., Boussard, J. & Sahani, M. Learning interpretable continuous-time models of latent stochastic dynamical systems. In *Proceedings of the 36th International Conference on Machine Learning*, vol. 97 of *Proceedings of Machine Learning Research*, 1726–1734 (2019).

[24] Kim, T. D. *et al.* Flow-field inference from neural data using deep recurrent networks. bioRxiv preprint at https://www.biorxiv.org/content/10.1101/2023.11.14.567136v1 (2023).

[25] Genkin, M. & Engel, T. A. Moving beyond generalization to accurate interpretation of flexible models. *Nat. Mach. Intell.* **2**, 674–683 (2020).

[26] Belkin, M., Hsu, D., Ma, S. & Mandal, S. Reconciling modern machine-learning practice and the classical bias–variance trade-off. *Proc. Natl. Acad. Sci. U.S.A.* **116**, 15849–15854 (2019).

[27] Wang, J., Tsin, D. & Engel, T. Predictive variational autoencoder for learning robust representations of time-series data. Workshop UniReps: Unifying Representations in Neural Models. In *Advances in Neural Information Processing Systems* (2023).

[28] Gao, Y., Archer, E., Paninski, L. & Cunningham, J. P. Linear dynamical neural population models through nonlinear embeddings. In *Advances in Neural Information Processing Systems* (2016).

[29] Wu, A., Roy, N. G., Keeley, S. & Pillow, J. W. Gaussian process based nonlinear latent structure discovery in multivariate spike train data. In *Advances in Neural Information Processing Systems* (2017).

[30] Bishop. *Pattern Recognition and Machine Learning* (Springer, New York, 2007).

[31] Wilcox, R. M. Exponential operators and parameter differentiation in quantum physics. *J. Math. Phys.* **8**, 962–982 (1967).

[32] Haas, K. R., Yang, H. & Chu, J.-W. Trajectory entropy of continuous stochastic processes at equilibrium. *J. Phys. Chem. Lett.* **5**, 999–1003 (2014).

4 Supplementary Figures

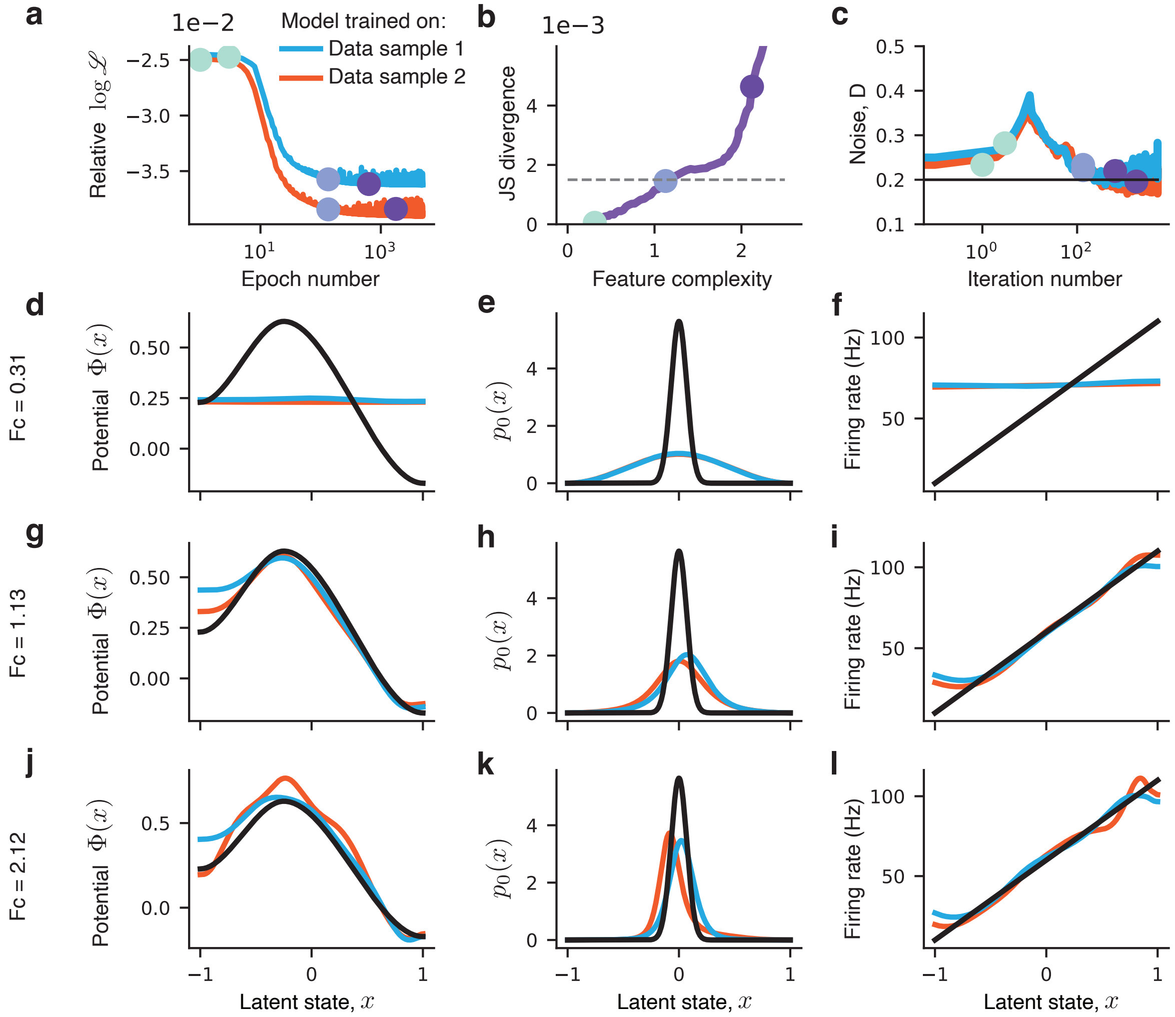

Supplementary Figure 1. Flexible inference and model selection on synthetic data with known ground truth. We use 400 trials of synthetic spike data for a single neuron generated from a ground-truth model with a single-barrier potential, narrow zero-centered $p_0(x)$ distribution, and linear tuning function. **a**, We fit our model independently on two halves of the data $\mathcal{D}_1$ and $\mathcal{D}_2$ (200 trials each). Relative log-likelihood ($[\log \mathscr{L}_0 - \log \mathscr{L}] / \log \mathscr{L}_0$, where $\mathscr{L}_0$ is the model likelihood at the initialization) decreases with the epoch number while the model discovers features of increasing complexity. **b**, The standard practice of selecting the model based on the epoch with the highest validated likelihood often yields fits with spurious features that cannot be reliably interpreted [25]. We developed an alternative strategy for identifying models with correct interpretation based on the consistency of features discovered from different data samples to separate true features from noise. We use Jensen-Shannon divergence (JS, y-axis) to compare features between the models with the same feature complexity (FC, x-axis) inferred from two data halves. Colored dots indicate three levels of FC that illustrate underfitting (mint), optimal fit (blue), and overfitting (purple) regimes. The optimal model is selected at FC where JS divergence exceeds a fixed threshold $\text{JS}_{\text{thres}} = 0.0015$, which was the same for all results in this study. **c**, Noise magnitude D for each training epoch for models fitted on $\mathcal{D}_1$ and $\mathcal{D}_2$. Black line indicates the ground-truth value of D. **d**, The ground-truth potential (black) and the potentials of the models fitted on $\mathcal{D}_1$ and $\mathcal{D}_2$ (colored lines) for low feature complexity (mint dot in b). The inferred potentials overlap but miss some of the ground-truth features, which is the underfitting regime. **e**, Same as d for the initial state distribution $p_0(x)$. **f**, Same as d for the tuning function $f(x)$. **g-i**, Same as d-f for the optimal feature complexity (blue dot in b). The models inferred from two data halves overlap and match the ground-truth model. **j-l**, Same as d-f for high feature complexity (purple dot in b). The models inferred from two data halves diverge and disagree with the ground truth, which is the overfitting regime.

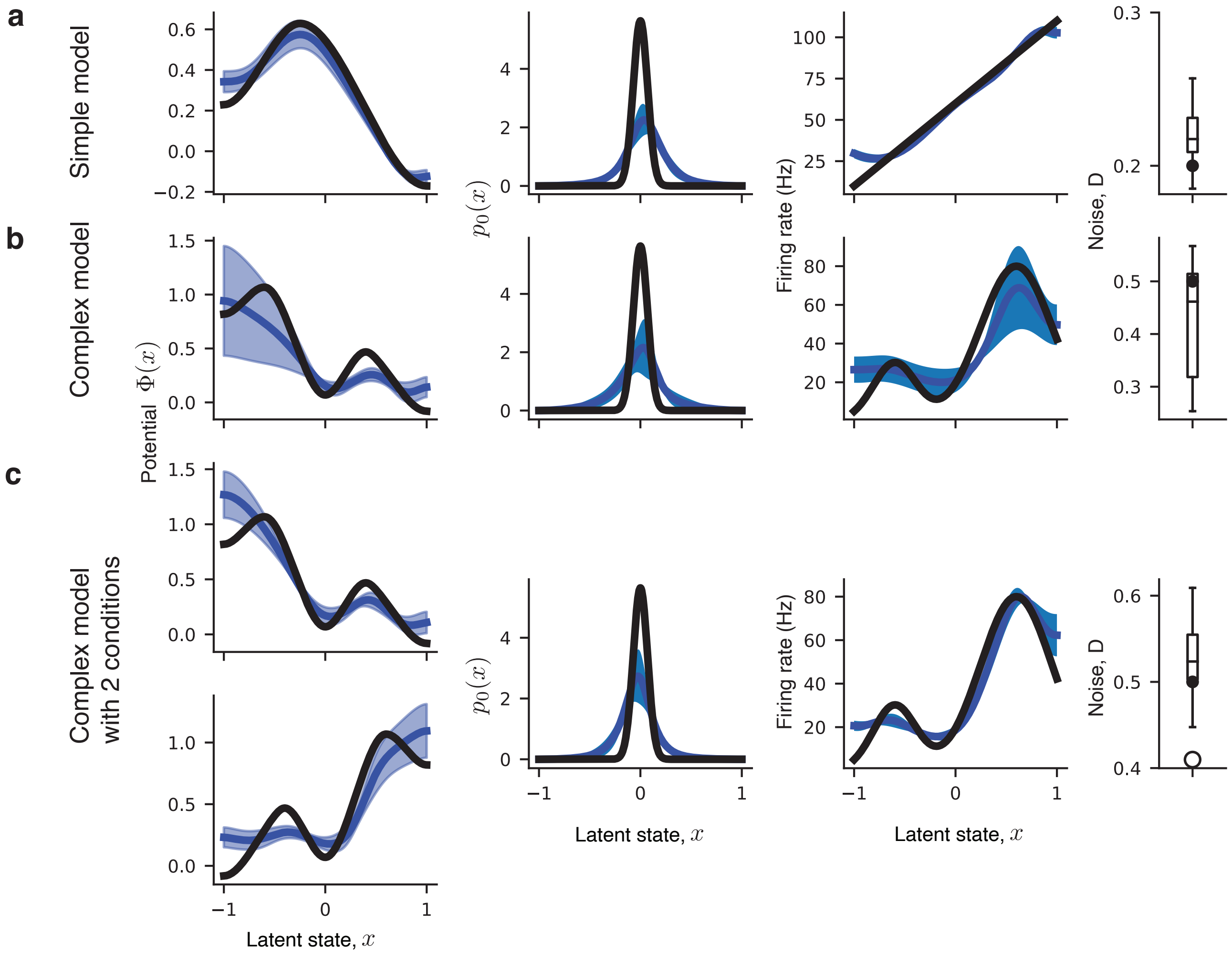

Supplementary Figure 2. Inference of dynamics with different complexity from synthetic single-neuron data. We generate spike data for a single neuron using our model with varying complexity of the potential and tuning function, with the amount of data (800 trials total) similar to our PMd recordings. We perform the model fitting and model selection to test how the inference accuracy depends on the complexity of the ground-truth dynamics. **a**, The inferred potential (left), $p_0(x)$ distribution (middle left), tuning function (middle right) and noise magnitude (right) for a model with a single-barrier potential and a linear tuning function. The inferred model (blue) tightly overlaps with the ground truth (black). **b**, Same as a for a model that has a more complex potential with two barriers and a non-linear tuning function. The inferred model matches the ground truth, but the estimation uncertainty is higher and the inference is less accurate in the regions that are poorly sampled by the dynamics in the data (the region with high potential near the left boundary). The inference accuracy improves when fitting data from a population of neurons (Supplementary Fig. 3). **c**, Shared optimization across two stimulus conditions for the same model as in b. In two conditions, the potentials are mirror images of each other, and $p_0(x)$, D and tuning function are the same. In the shared optimization, we restrict $p_0(x)$, D and the tuning function to be the same across conditions. The shared optimization leads to a lower estimation uncertainty and more accurate inference (cf. to b). The inference accuracy further improves when fitting data from a population of neurons using shared optimization (Supplementary Fig. 4). In all panels, error bars are s.t.d. over 10 bootstrap samples.

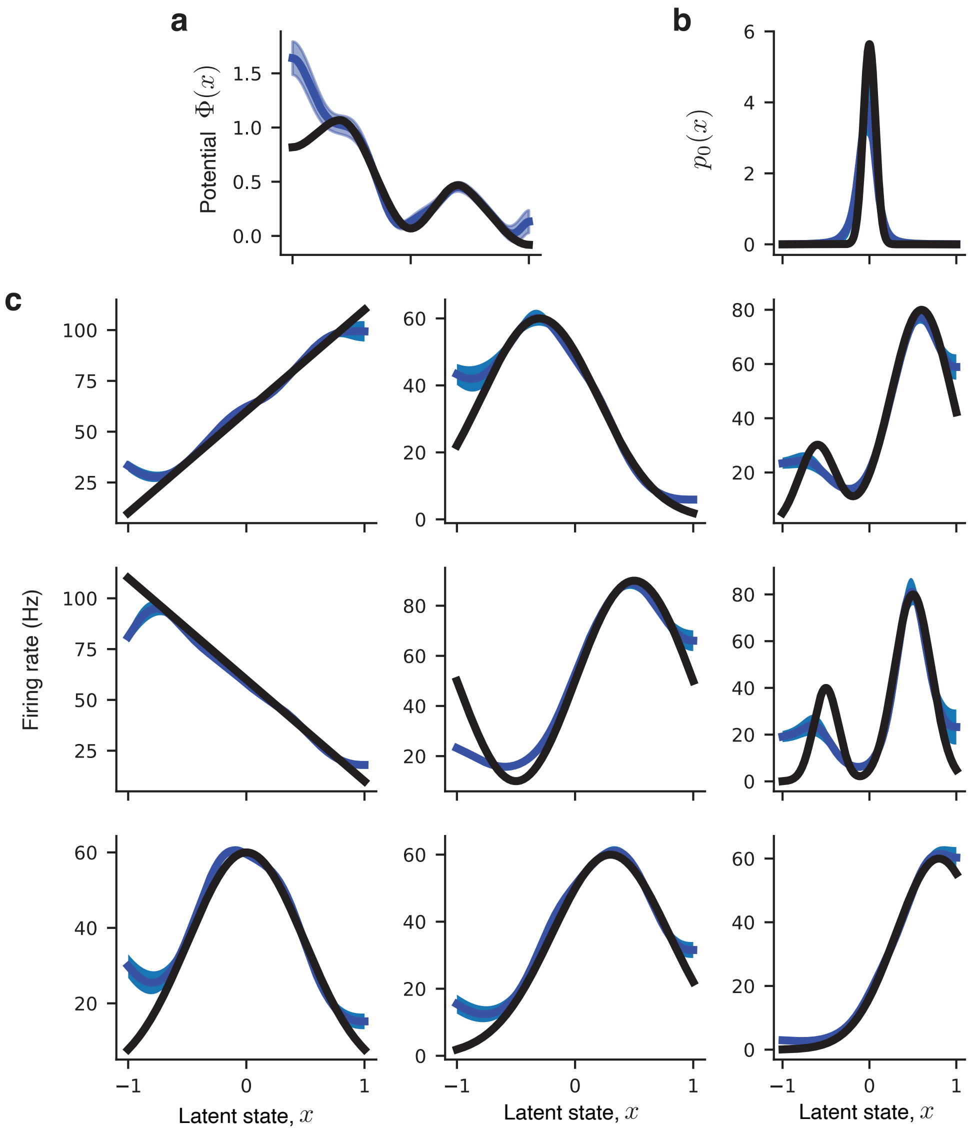

Supplementary Figure 3. Inference of complex dynamics improves on synthetic population data relative to single-neuron data. We generate spike data for a population of 9 neurons using our model with a complex two-barrier potential (same is in Supplementary Fig. 2b) and heterogeneous non-linear tuning functions, with the amount of data (800 trials total) similar to our PMd recordings. We fit these data with the population model to test the inference accuracy. **a**, The inferred potential (blue) tightly overlaps with the ground truth (black). The inference is inaccurate near the left boundary, since this region with the high potential is poorly sampled by the dynamics in the data. The inference accuracy further improves for the shared optimization across multiple stimulus conditions (Supplementary Fig. 4). **b**, The inferred $p_0(x)$ distribution (blue) tightly overlaps with the ground truth (black). **c**, The inferred tuning functions (blue) tightly overlap with the ground truth (black). The inference of tuning functions is more accurate compared to the single-neuron models (cf. Supplementary Fig. 2), because spikes of multiple neurons in the population contribute to a more precise estimation of the latent state, which in turn enables a more accurate inference of the tuning function of each neuron. In all panels, error bars are s.t.d. over 10 bootstrap samples.

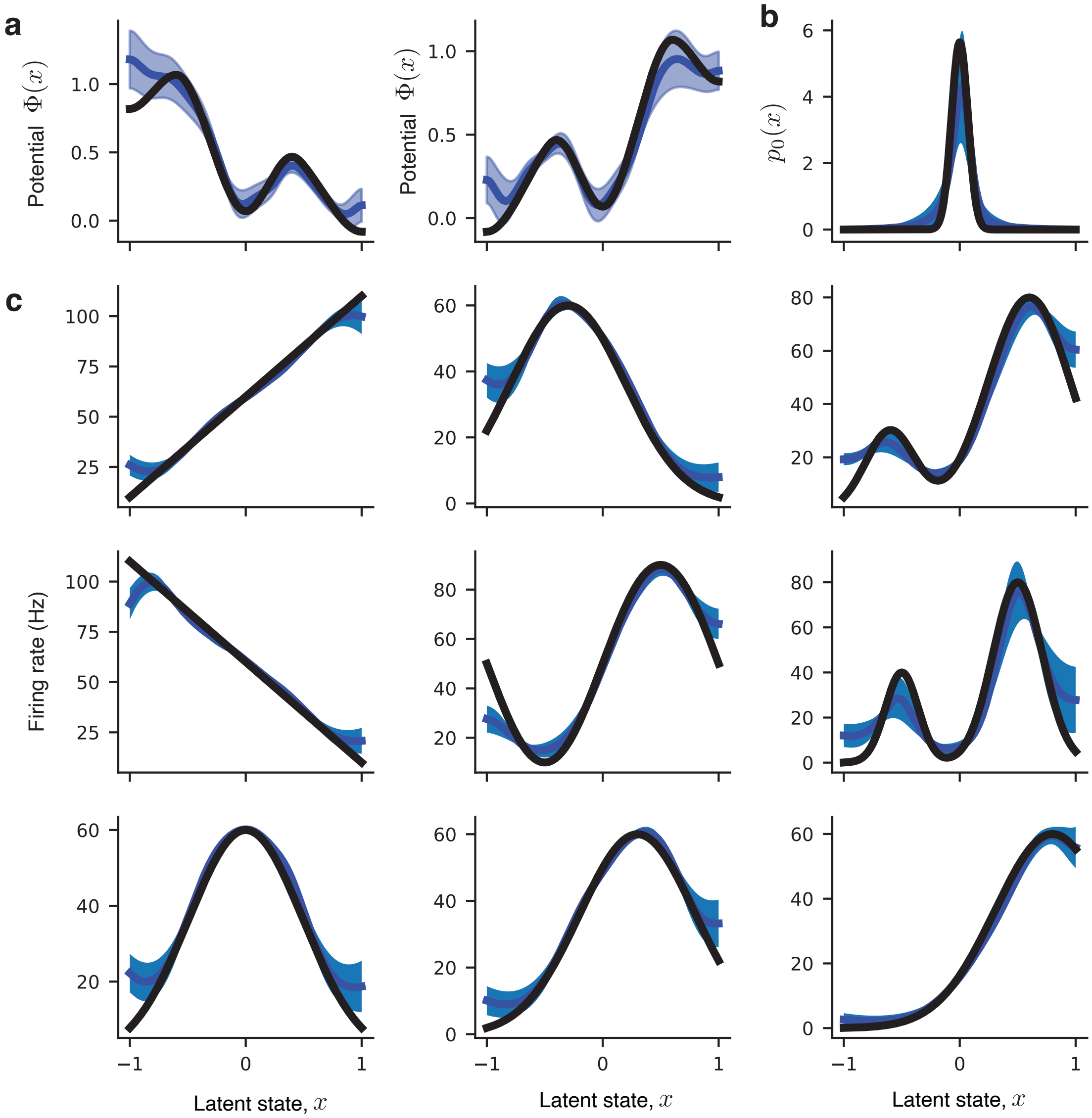

Supplementary Figure 4. Shared optimization enables accurate inference of complex dynamics and tuning functions in synthetic population data. We generate spike data for a population of 9 neurons using the same model as in Supplementary Fig. 3 for two stimulus conditions. In two conditions, the potentials are mirror images of each other, and $p_0(x)$, D and tuning function are the same. In the shared optimization, we restrict $p_0(x)$, D and the tuning functions to be the same across conditions. We fit the shared population model to test the inference accuracy. **a**, The inferred potentials (blue) tightly overlap with the ground truth (black) in both conditions. The inference is accurate even in the regions with high potential in each condition, because the tuning functions inferred from one condition enable more accurate inference of dynamics from limited data in the other condition. **b**, The inferred $p_0(x)$ distribution (blue) tightly overlaps with the ground truth (black). **c**, The inferred tuning functions (blue) tightly overlap with the ground truth (black). The inference is accurate on both left and right sides of the latent space, since the regions poorly sampled by the dynamics in one condition are sampled well in another condition, which enables the accurate inference of tuning functions in the entire domain. In all panels, error bars are s.t.d. over 10 bootstrap samples.

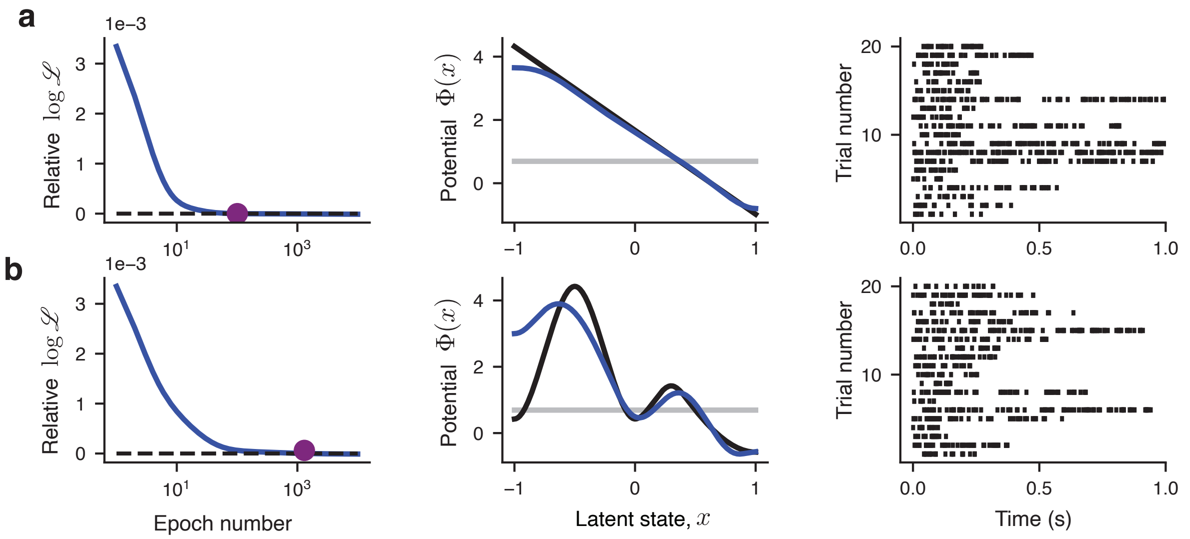

Supplementary Figure 5. Our nonparametric model correctly identifies ramping and stepping dynamics in synthetic data. a, The ramping model of decision-making assumes that on single trials neural activity evolves gradually towards a decision boundary as a linear drift-diffusion process, which corresponds to a linear potential with a constant slope in our modeling framework. We generated 1,600 trials of spike data for a single neuron (*right*, 20 example trials shown) with the ramping dynamics defined by a linear potential (*center*, black line) and linear tuning function to the latent variable. We fitted our single-neuron model to these data. Relative log-likelihood (defined as $[\log \mathscr{L}_{\mathrm{gt}} - \log \mathscr{L}]/\log \mathscr{L}_{\mathrm{gt}}$, where $\log \mathscr{L}_{\mathrm{gt}}$ is the log-likelihood of the ground-truth model) decreases with the epoch number (*left*). When the relative log-likelihood is zero (black dashed line), the likelihoods of the fitted and ground-truth models are equal (purple point). At this epoch, the inferred potential closely matches the ground-truth potential (*center*, blue line – fitted potential on the epoch when the relative log-likelihood is zero, grey line – initialization, black line – ground truth). **b**, Same as a for the stepping model. The stepping model assumes that on single trials neural activity abruptly jumps from the initial to a final state representing a choice, which corresponds to a potential with two barriers where trajectories have to overcome one of the barriers to reach either decision boundary.

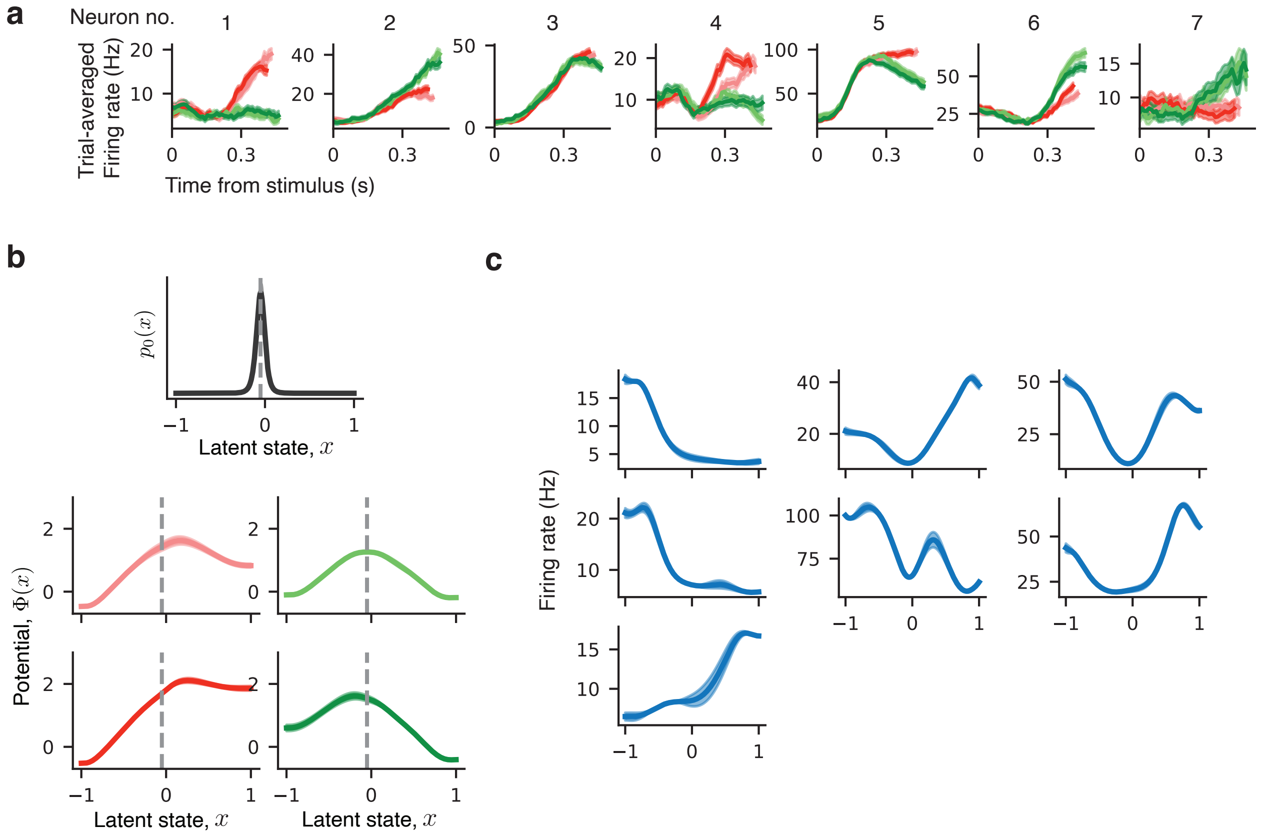

Supplementary Figure 6. The inferred model with a single-barrier potential for additional example population of PMd neurons. a, Trial-averaged firing rates sorted by the chosen side and stimulus difficulty for a population of 7 neurons recorded simultaneously from monkey O. Error bars are s.e.m. over trials. **b**, The inferred potentials for four stimulus conditions (middle and lower panels) and $p_0(x)$ distribution shared across conditions (upper panel) for the population in a. **c**, The inferred tuning functions shared across conditions for the population in a. Error bars in panels b,c are s.t.d. over 10 bootstrap samples.

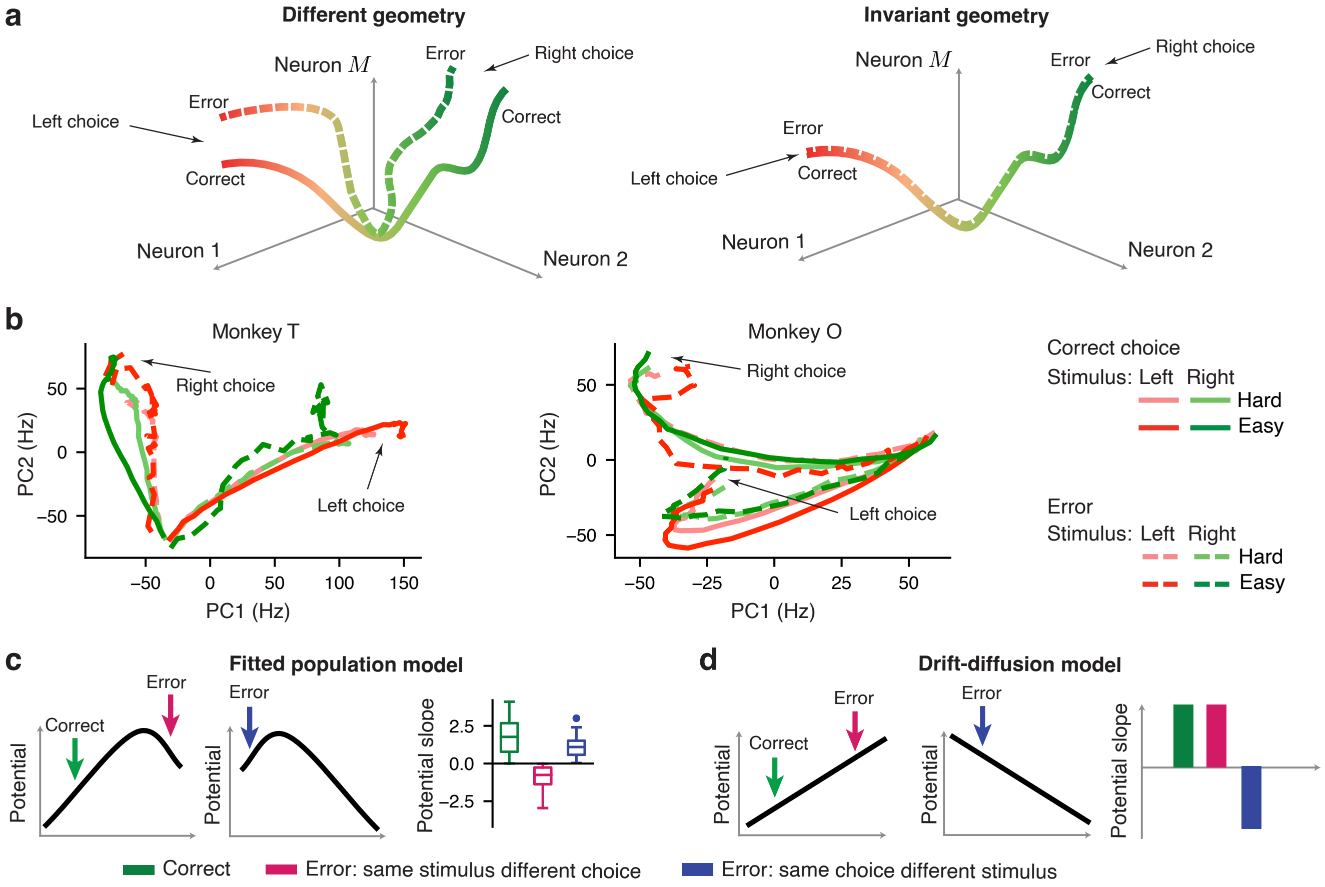

Supplementary Figure 7. Distinct population dynamics with invariant geometry on correct versus error trials. **a**, Our model allows us to test how errors arise in single-trial population activity. One hypothesis (left) is that single-trial trajectories take distinct paths through the population state space on correct (solid line) versus error trials (dashed line), each along a separate one-dimensional manifold. An alternative hypothesis (right) is that all trials leading to the same choice—whether correct or error—unfold along the same manifold, implying that the geometry of the choice manifold remains invariant between correct decisions (solid line) and errors (dashed line). **b**, The geometry of trial-averaged trajectories cannot distinguish between these alternatives, because trial-averaged responses conflate the dynamics and geometry of single-trial representations (Extended Data Fig. 3). In our PMd data, trial-averaged trajectories leading to the same choice are distinct between correct (solid lines) and error trials (dashed lines). Trial-averaged population responses projected onto the first two principal components are shown for all successfully fitted neurons (monkey T: $n = 117$, monkey O: $n = 67$), sorted by the stimulus side, difficulty, and whether the choice was correct (solid lines) or error (dashed lines). Differences in trial-averaged trajectories suggest that the choice manifold may differ between correct and error decisions (as in the hypothesis shown on the left in panel a). In contrast, our model revealed that tuning functions were invariant across stimulus conditions (Extended Data Fig. 7), supporting the "invariant geometry" hypothesis (shown on the right in panel a). **c**, While geometry of choice representation in PMd was invariant on correct and error trials, the dynamics were distinct. The flow field drives the dynamics in opposite directions along the manifold on correct versus error trials for the same stimulus, as indicated by the different sign of the potential slope (green vs. red arrow). Moreover, the dynamics leading to the same choice evolve faster when this choice is correct than error, as indicated by a steeper slope of the potential on the side corresponding to the correct choice (green vs. blue arrow). The box plot shows the potential slope for all successfully fitted neurons ($n = 184$), evaluated on the side corresponding to the correct choice (green), the error choice for the same stimulus (red), and the error choice for the opposite stimulus that results in the same decision (blue). Center lines indicate medians; boxes span the 25th to 75th percentiles; whiskers extend to the nearest of 1.5× the interquartile range or the most extreme data point; outliers beyond the whiskers are shown as dots. **d**, In contrast to dynamics in PMd, in the drift-diffusion model (Supplementary Fig. 5a), the flow field drives the dynamics towards the correct-choice boundary on both correct (green) and error trials (red), and the potential slope is the same for correct decisions (green) and errors (blue).

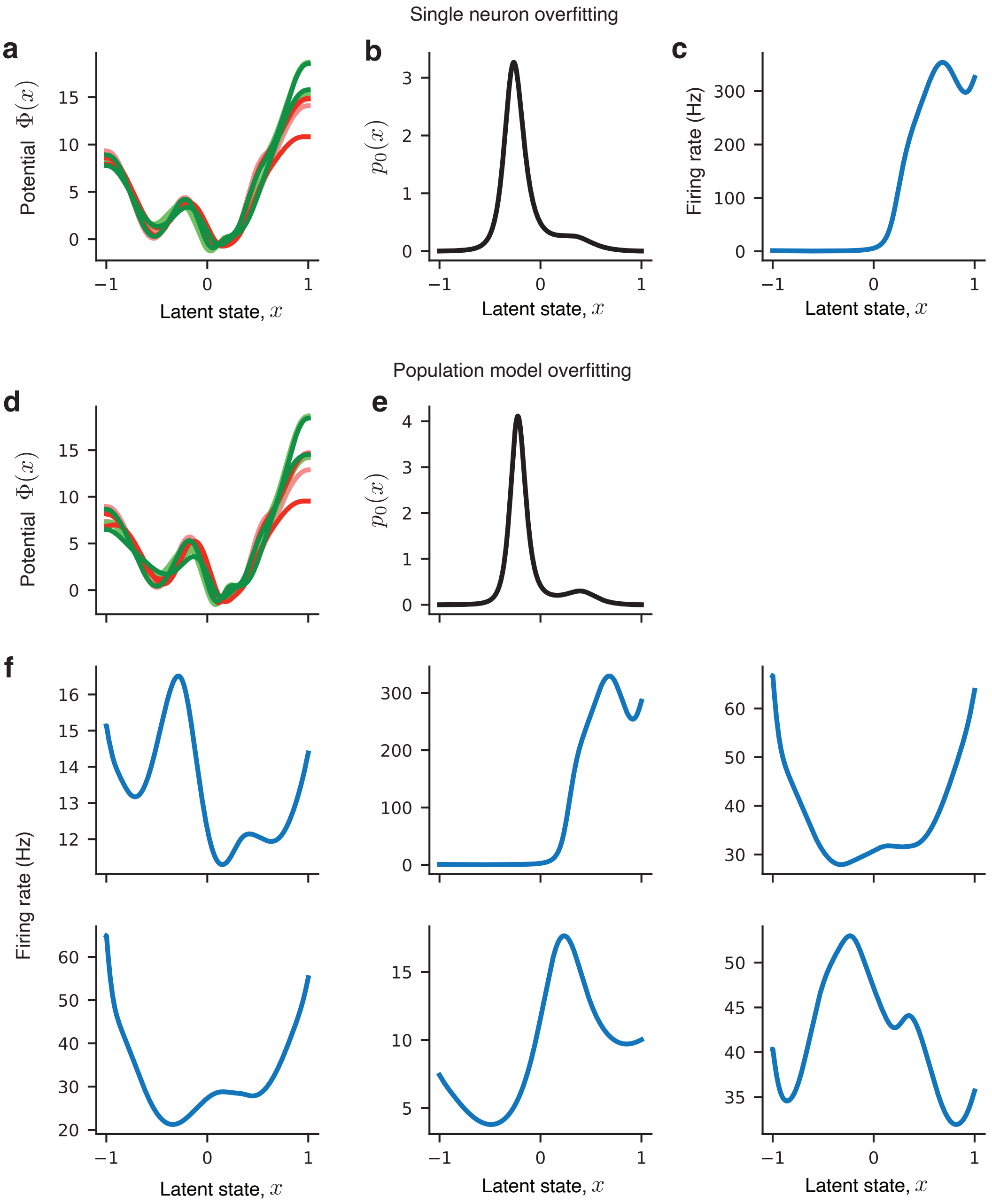

Supplementary Figure 8. Instances of overfitting in PMd data. When fitting our model to PMd data and performing model selection, we observed three instances of overfitting: 1 single neuron and 1 population from monkey O, and 1 population from monkey T. **a**, The model showing overfitting for the single neuron from monkey O. The inferred potentials are the same across four stimulus conditions and show deep wells compensated by a disproportionally high noise magnitude ($D \sim 3-5$, compared to $D \sim 0.2-0.6$ in regular fits). This model produces severely underestimated reaction times (reaction time ∼10 ms in the model, compared to ∼500 ms in the data) and does not predict monkey's choice. **b**, The inferred $p_0(x)$ shared across conditions for the model in a. **c**, The inferred tuning function shared across conditions for the model in a shows unrealistically high firing rates up to several hundreds of Hz. **d**, The model showing overfitting for the population from monkey O. The inferred potentials are the same across four stimulus conditions and show deep wells compensated by a disproportionally high noise magnitude ($D \sim 4$). This model produces severely underestimated reaction times and does not predict monkey's choice. The overfitted model for the population from monkey T had similar features (not shown here). **e**, The inferred $p_0(x)$ shared across conditions for the model in d. **f**, The inferred tuning functions shared across conditions for the model in d. The single neuron shown in a-c is part of this population (upper row, middle), and its tuning function shows unrealistically high firing rates in the population model as well.

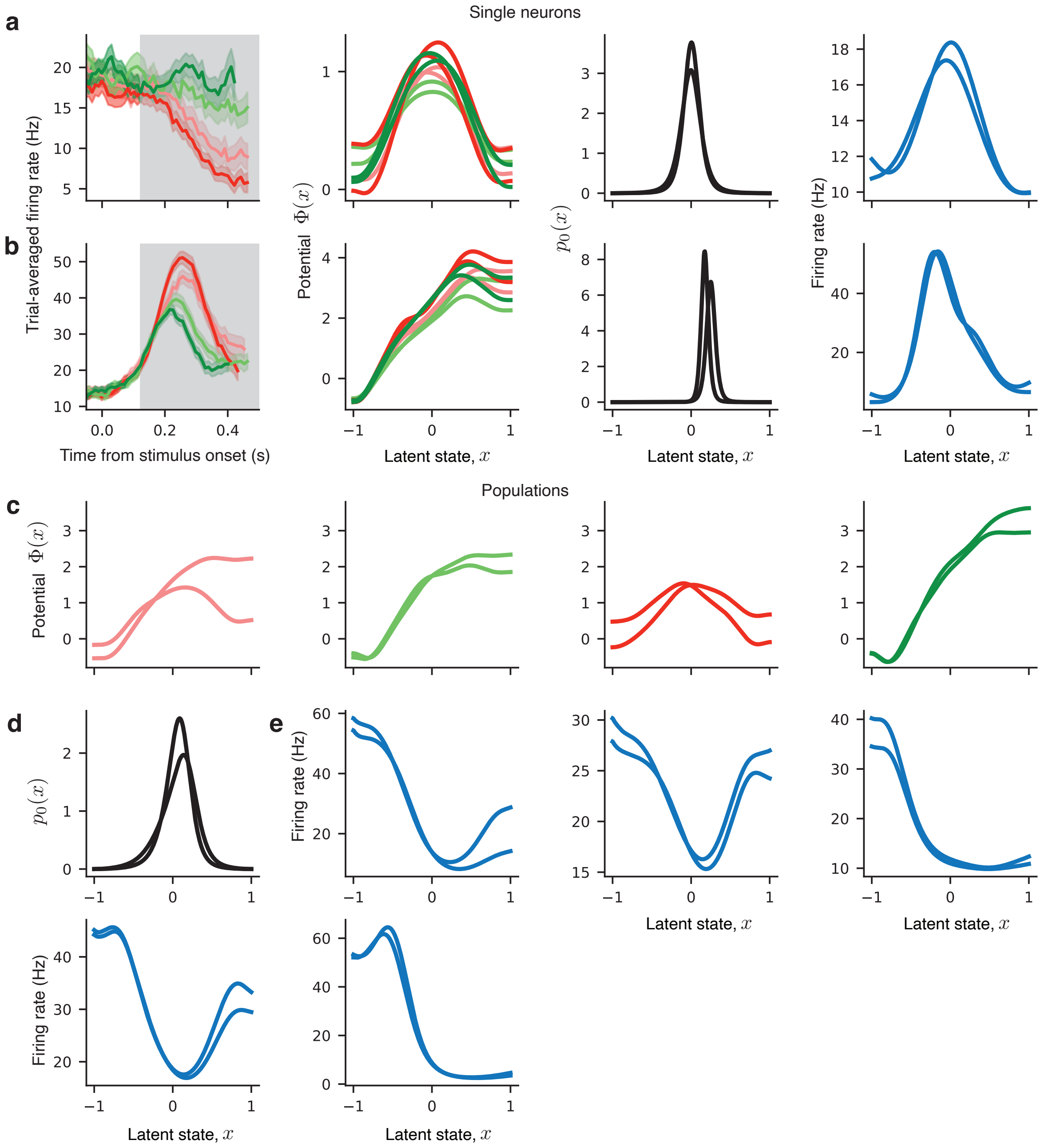

Supplementary Figure 9. Types of underfitting in PMd data. We observed two types of underfitting: no decision signal (a,b) and disagreement between data splits (c-e), both of which may arise when a model cannot detect a weak decision signal and mainly fits the condition-independent trend in neural activity. **a**, A model showing underfitting for an example single neuron. Left to right: trial-averaged firing rates sorted by the chosen side and stimulus difficulty, the inferred potentials for four stimulus conditions, $p_0(x)$ and the tuning function shared across conditions. The potentials have a similar symmetric shape across all conditions, such that the left and right choices are equally probable, and the tuning function is also symmetric. This model captures only the overall ramping trend but not the decision signal, which likely results from weak choice selectivity of this neuron. **b**, Same as a for another example neuron. In all conditions, the potentials point to the left boundary, predicting more left choices in all conditions. This model captures the speed of the dynamics (steeper slope for easy conditions) but not the decision signal. **c**, A model showing underfitting for an example population of 6 neurons. The potentials inferred from two data halves in four stimulus conditions. In easy-left condition (third column), the potentials disagree between the data splits pointing to the opposite boundaries, which leads to an early crossing of JS divergence threshold and the selection of a model with low feature complexity before all consistent features have been discovered. **d**, The inferred $p_0(x)$ shared across conditions for the model in c. **e**, The inferred tuning functions shared across conditions for the model in c.
