# Supplementary material for: The dynamics and geometry of choice in the premotor cortex
Source: Nature. 2025 Jun 25;645(8079):168–76. doi: 10.1038/s41586-025-09199-1 (PMC12408350; doi:10.1038/s41586-025-09199-1)
Supplement: Supplementary file 2 — Reporting Summary [file 41586_2025_9199_MOESM2_ESM.pdf]

nature portfolio

Corresponding author(s): Tatiana Engel

Last updated by author(s): May 15, 2025

Reporting Summary

Nature Portfolio wishes to improve the reproducibility of the work that we publish. This form provides structure for consistency and transparency in reporting. For further information on Nature Portfolio policies, see our Editorial Policies and the Editorial Policy Checklist.

Statistics

For all statistical analyses, confirm that the following items are present in the figure legend, table legend, main text, or Methods section.

n/a	Confirmed	
☐	☒	The exact sample size (*n*) for each experimental group/condition, given as a discrete number and unit of measurement
☐	☒	A statement on whether measurements were taken from distinct samples or whether the same sample was measured repeatedly
☐	☒	The statistical test(s) used AND whether they are one- or two-sided<br>*Only common tests should be described solely by name; describe more complex techniques in the Methods section.*
☐	☒	A description of all covariates tested
☒	☐	A description of any assumptions or corrections, such as tests of normality and adjustment for multiple comparisons
☐	☒	A full description of the statistical parameters including central tendency (e.g. means) or other basic estimates (e.g. regression coefficient) AND variation (e.g. standard deviation) or associated estimates of uncertainty (e.g. confidence intervals)
☐	☒	For null hypothesis testing, the test statistic (e.g. *F*, *t*, *r*) with confidence intervals, effect sizes, degrees of freedom and *P* value noted<br>*Give P values as exact values whenever suitable.*
☒	☐	For Bayesian analysis, information on the choice of priors and Markov chain Monte Carlo settings
☒	☐	For hierarchical and complex designs, identification of the appropriate level for tests and full reporting of outcomes
☐	☒	Estimates of effect sizes (e.g. Cohen's *d*, Pearson's *r*), indicating how they were calculated

Our web collection on statistics for biologists contains articles on many of the points above.

Software and code

Policy information about availability of computer code

Data collection	Mathworks xPC Target 5.3<br>MATLAB R2012b<br>Psychtoolbox Version 3.0.9<br>Central version 6.0<br>Matclust version 1.7.0.0 (2015)<br>Plexon Offline Sorter v3
Data analysis	Data analysis was performed using custom Python 3 code. The source code to reproduce the analyses is available as the NeuralFlow Python package on GitHub (https://github.com/engellab/neuralflow) and archived on Zenodo (https://doi.org/10.5281/zenodo.15426288).

For manuscripts utilizing custom algorithms or software that are central to the research but not yet described in published literature, software must be made available to editors and reviewers. We strongly encourage code deposition in a community repository (e.g. GitHub). See the Nature Portfolio guidelines for submitting code & software for further information.

Data

Policy information about availability of data

All manuscripts must include a data availability statement. This statement should provide the following information, where applicable:

- Accession codes, unique identifiers, or web links for publicly available datasets
- A description of any restrictions on data availability
- For clinical datasets or third party data, please ensure that the statement adheres to our policy

Neural recording data are available on Figshare at https://doi.org/10.6084/m9.figshare.29052116.v1. The synthetic data used in this study can be reproduced using the source code.

Research involving human participants, their data, or biological material

Policy information about studies with human participants or human data. See also policy information about sex, gender (identity/presentation), and sexual orientation and race, ethnicity and racism.

Reporting on sex and gender	NA
Reporting on race, ethnicity, or other socially relevant groupings	NA
Population characteristics	NA
Recruitment	NA
Ethics oversight	NA

Note that full information on the approval of the study protocol must also be provided in the manuscript.

Field-specific reporting

Please select the one below that is the best fit for your research. If you are not sure, read the appropriate sections before making your selection.

☒ Life sciences ☐ Behavioural & social sciences ☐ Ecological, evolutionary & environmental sciences

For a reference copy of the document with all sections, see nature.com/documents/nr-reporting-summary-flat.pdf

Life sciences study design

All studies must disclose on these points even when the disclosure is negative.

Sample size	The sample sizes used in this study are consistent with standard practices in systems neuroscience involving non-human primates, e.g., Mante et al. Nature 503, 78–84 (2013); Engel et al. Science 354,1140–1144 (2016); Peixoto et al. Nature 80, 791–21 (2021); Steinemann et al. eLife 12, RP90859 (2024). Data were collected from two macaque monkeys (monkey T: 75 sessions; monkey O: 66 sessions), providing a robust number of sessions per animal. No statistical methods were used to predetermine sample size.
Data exclusions	No data were excluded in this study.
Replication	All key findings were replicated in both monkeys.
Randomization	Participants were not allocated into groups. Subject randomization is not relevant to this study. Both animals performed the same task under the same conditions, over many sessions and trials. All trials were randomized as were placements of electrodes on a day to day basis.
Blinding	Blinding is not relevant to this study as the macaques were not placed into different groups, they performed the same task under the same conditions from session to session.

Reporting for specific materials, systems and methods

We require information from authors about some types of materials, experimental systems and methods used in many studies. Here, indicate whether each material, system or method listed is relevant to your study. If you are not sure if a list item applies to your research, read the appropriate section before selecting a response.

Materials & experimental systems

n/a	Involved in the study
☒	☐ Antibodies
☒	☐ Eukaryotic cell lines
☒	☐ Palaeontology and archaeology
☐	☒ Animals and other organisms
☒	☐ Clinical data
☒	☐ Dual use research of concern
☒	☐ Plants

Methods

n/a	Involved in the study
☒	☐ ChIP-seq
☒	☐ Flow cytometry
☒	☐ MRI-based neuroimaging

Animals and other research organisms

Policy information about studies involving animals; ARRIVE guidelines recommended for reporting animal research, and Sex and Gender in Research

Laboratory animals	2 male macaque mulatta. T – 7 years old, O – 11 years old.
Wild animals	The study did not involve wild animals.
Reporting on sex	Both macaques were males. Female macaque monkeys are difficult to obtain because of their use for breeding. The small number of animals used in primate electrophysiology studies precludes any statements about sex differences.
Field-collected samples	The study did not involve samples collected from the field.
Ethics oversight	Stanford University Institutional Animal Care and Use Committee approved the study protocol (8856).

Note that full information on the approval of the study protocol must also be provided in the manuscript.

Plants

Seed stocks	NA
Novel plant genotypes	NA
Authentication	NA
